# Synthesis, antimicrobial activity and molecular docking studies of spiroquinoline-indoline-dione and spiropyrazolo-indoline-dione derivatives

**DOI:** 10.1038/s41598-023-27777-z

**Published:** 2023-01-30

**Authors:** Melek Gul, Emine Turk Celikoglu, Onder Idil, Gamze Tas, Emel Pelit

**Affiliations:** 1 Department of Chemistry, Faculty of Art and Sciences, Amasya University, 05100 Amasya, Turkey; 2 Department of Biology, Faculty of Art and Sciences, Amasya University, 05100 Amasya, Turkey; 3 Department of Pre-School Education, Faculty of Education, Amasya University, 05100 Amasya, Turkey; 4 Department of Chemistry, Faculty of Art and Sciences, Kirklareli University, 39100 Kirklareli, Turkey

**Keywords:** Chemistry, Organic chemistry, Chemical synthesis

## Abstract

Spiro[benzo[h]quinoline-7,3′-indoline]diones and spiro[indoline-3,4′-pyrazolo[3,4-b]quinoline]diones were efficiently synthesized via one-pot multi-component reactions under ultrasound-promoted conditions. Spiro[benzo[h]quinoline-7,3′-indoline]dione derivatives were successfully developed by the reaction of isatins, naphthalene-1-amine and 1,3-dicarbonyl compounds. The spiro[indoline-3,4′-pyrazolo[3,4-b]quinoline]dione derivatives were prepared by the reaction of isatins, 5-amino-1-methyl-3-pheylpyrazole, and 1,3-dicarbonyl compounds by using ( ±)-camphor-10-sulfonic acid as a catalyst in H_2_O/EtOH (3:1 v/v) solvent mixture. The antibacterial activity of the synthesized compounds was evaluated against, *Enterococcus faecalis*, *Staphylococcus aureus* and *Candida albicans*. Compounds 4b, 4h, and 6h showed the strongest antimicrobial activity toward both bacteria. The MIC values of these compounds ranged from 375–3000 µg/mL. The effect of these compounds (4b, 4h, 6h) as a function of applied dose and time was investigated by a kinetic study, and the interaction with these antimicrobial results was simulated by a molecular docking study. We also used the docking approach with Covid-19 since secondary bacterial infections. Docking showed that indoline-quinoline hybrid compounds 4b and 4h exerted the strongest docking binding value against the active sites of 6LU7. In addition, the synthesized compounds had a moderate to good free radical scavenging activity.

## Introduction

Multicomponent reactions are valuable organic reactions in which three or more reactants react in a one-pot process to produce a final product^1^. These reactions have tremendous efficiency, especially for the synthesis of heterocyclic compounds that exhibit a wide range of biological activities^2^. Multicomponent reactions comply with the principles of green chemistry in terms of a high degree of atom economy, easier progress of reactions, low energy consumption, short reaction times, and lack of waste products^3^.

Quinoline and indole are importat moieties of a large number of natural products and biologically active compounds^4–7^. Quinoline derivatives were found to have anticancer^8^, anti-HIV^9^, antibacterial^10^, antimalarial^11^, anti-inflammatory^12^ activities. Indole derivatives also exhibit antimicrobial^13^, antibacterial^14^, anti-inflammatory^15^, antiviral^16^, antidiabetic^17^, antitumor^18^, and anticancer^19^ activities. The spiro-fuced oxindole moiety is also a significant core structure of many pharmacological agents and natural products^20–23^ and additionally has also been shown to be a potential fluorescent materials^24,25^. On the other hand heterocylic compounds containing a pyrazole moiety exhibit anti-HIV^26^, anticancer^27,28^, antifungal^29^, and antimicrobial^30–32^ activities. Due to the broad biological activities of these spiro-fuced oxindole and pyrazole derivatives many synthetic methods for their preparation have been described^33–43^. In addition, various catalysts such as HOAc^44^, PTSA^45^, CAN^46^, L-Proline^47^, [NMP]H_2_PO_4_^48^, ChCl/Lac^49^, Papain^50^, and Cu(OTf)_2_^51^ have been used to promote this reaction.

The improvement of diverse synthetic processes that provide better environmental performance in synthetic organic chemistry is an objective^52^. Ultrasound irradiation promotes many organic reactions, owing to cavitational collapse. Various organic reactions can be effectively accomplished in higher yields, with shorter reaction times, and under milder reaction conditions using ultrasonic irradiation^53–56^.

(±)-Camphor-10-sulfonic acid (CSA) is an effective, water-soluble and reusable organocatalyst that has been used in various organic reactions. For instance, Friedel–Crafts reactions^57^, the synthesis of dioxabicyclo[3.3.1]nonane^58^, and the synthesis of spirocyclic compounds^59^.

**Figure 1:**
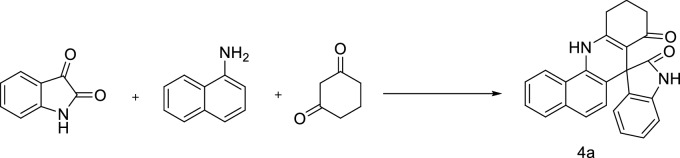
Synthesis of compound 4a.

**Figure 2:**
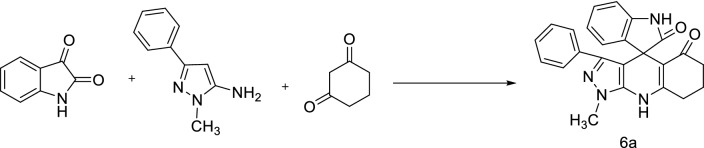
Synthesis of compound 6a.

The aim of this work is to obtain spiro[benzo[h]quinoline-7,3′-indoline]diones and spiro[indoline-3,4′-pyrazolo[3,4-b]quinoline]diones under ultrasonic irradiation in the presence of ( ±)-CSA catalyst in a H_2_O/EtOH solvent mixture (Figs. 1, 2).

To extend the biological curiosity of the synthesized compounds, they were tested for their antibacterial activity against *E. facealis*, *S. aureus*, and *C. albicans*. The radical scavenging activity (determined by DPPH) of these compounds was also investigated. In general, effective HAT agents are compounds that have a high hydrogen atom donating ability and low heteroatom H-bond dissociation energy. Removal of hydrogen from these compounds results in C-centered radicals that are stabilized by resonance or the formation of sterically hindered radicals^60^. Co-infections in covid 19 pose a high risk, especially in intensive care units^61^. In this molecular docking study, *S. aureus* related biotin protein ligase, *E. Facealis* related alanine racemase, and protease N3 complex proteins of covid 19 were selected. In addition, molecular docking simulations were given chractersitics to show their mode of binding energy and the affection of these compounds against Sar-Cov-2 main protease inhibition^62^, *Staphylococcus aureus* biotin-protein ligase inhibitor^63^, and *E.faecalis* alanine racemase activity^64^.

## Results and discussion

### Chemistry

The three-component reaction of isatin (1.00 mmol), 1,3-cyclohexadione (1.00 mmol), and naphthalene-1-amine (1.00 mmol) was used as a model reaction to optimize the reaction conditions. In the presence of %5 mol CSA in H_2_O compound 4a was obtained in 60 min with a yield of 26% under ultrasonic irradiation at room temperature. When the same reaction was examined in the same conditions in a H_2_O/EtOH (3:1, v/v) solvent mixture, the product was obtained with a yield of 74% in 45 min (Table 1).

**Table 1:** Optimization of ( ±)-CSA catalyst loading in the synthesis of compound 4a.

( ±)-CSA (mol. %)	Temperature (°C)	Solvent	Conditions	Time (min)	Yield (%)^a^
–	25	EtOH	Stirring	90	Trace
5	25	H_2_O	Stirring	90	Trace
5	25	H_2_O	US	60	26
5	25	H_2_O/EtOH (3:1)	US	45	74
5	50	H_2_O/EtOH (3:1)	US	30	88
10	50	H_2_O/EtOH (3:1)	US	30	89

When the temperature was increased to 50 °C the product was obtained in 88% yield, and the reaction was completed in 30 min (Table 1). A series of spiro[benzo[*h*]quinoline-7,3′-indoline]diones were synthesized using the optimized reaction conditions under ultrasonic irradiation. The results are summarized in Table 2.

**Table 2:** Synthesis of spiroquinoline-indoline-dione (Spirooxindole) derivatives in the presence of catalyst ( ±)-CSA.


Entry	Isatin/R	1,3-dicarbonyl compounds	Product	Time (min)	Yield (%)
1	H	1,3-Cyclohexanedione	4a	30	88
2	H	5,5-Dimethyl-1,3-cyclohexadione	4b*	30	84
3	H	1,3-Cyclopentanedione	4c	30	85
4	H	1,3-Indandione	4d*	30	89
5	NO_2_	1,3-Cyclohexanedione	4e	30	85
6	NO_2_	5,5-Dimethyl-1,3-cyclohexadione	4f	30	87
7	NO_2_	1,3-Cyclopentanedione	4g	30	90
8	NO_2_	1,3-Indandione	4h*	30	92

*These compounds were synthesized by another method in the literature^44^.

Then the spiro[indoline-3,4′-pyrazolo[3,4-*b*]quinoline]diones were synthesized via multicomponent reaction of isatins, 5-amino-1-methyl-3-pheylpyrazole, and 1,3-dicarbonyl compounds in the presence of %5 mol ( ±)-CSA in H_2_O/EtOH (3:1, v/v) under ultrasonic irradiation at 50 °C (Table 3).

**Table 3:** Synthesis of spiropyrazolo-indoline-dione (Spirooxindole) derivatives in the presence of catalyst ( ±)-CSA.


Entry	Isatin/R	1,3-Dicarbonyl compounds	Product	Time (min)	Yield (%)
1	H	1,3-Cyclohexanedione	6a	30	84
2	H	5,5-Dimethyl-1,3-cyclohexadione	6b	30	87
3	H	1,3-Cyclopentanedione	6c	30	90
4	H	1,3-Indandione	6d	30	89
5	NO_2_	1,3-Cyclohexanedione	6e	30	86
6	NO_2_	5,5-Dimethyl-1,3-cyclohexadione	6f	30	85
7	NO_2_	1,3-Cyclopentanedione	6g	30	91
8	NO_2_	1,3-Indandione	6h	30	93

The structure of the synthesized compounds was confirmed by Fourier transform-infrared (FTIR), ^1^H NMR, ^13^C, NMR techniques and mass spectroscopy. The NH peaks were observed at 3600–3200 cm^−1^ and the C=O peaks were observed at 1730–1670 cm^-1^ in the FTIR spectra. In the ^1^H NMR spectra of compounds 4a–h and 6a–h, the aliphatic C–H protons resonated at *δ* 1.00–2.97 ppm and the aromatic C–H protons resonated at *δ* 6.40–8.75 ppm, and the N–H protons resonated t *δ* 9.35–11.40 ppm. In the ^13^C NMR spectra, the aliphatic carbons resonated at *δ* 18.50–53.00 ppm. The quareternery carbons in spiro moiety resonated at *δ* 47.00–55.00 ppm. The aromatic carbons resonated at *δ* 99.00–168.00 ppm. The carbonyl carbons resonated at *δ* 175.00–200.00 ppm. The mass spectra of all synthesized compounds exhibited the expected molecular ion peak.

### Antimicrobial activity

The increasing resistance of microorganisms to antibiotics makes it necessary to synthesize compounds that can be used as antibiotics. For this reason, the research of compounds with antimicrobial properties has gained momentum in recent years^65,66^. In this part of the study, the antimicrobial activities of the synthesized spiro[benzo[h]quinoline-7,3′-indoline]dione and spiro[indoline-3,4′-pyrazolo[3,4-b]quinoline]dione derivatives were investigated. For the antimicrobial studies, *E. faecalis* and *S. aureus* were used as bacteria and *C. albicans* as yeast samples. The stock solutions of all compounds used in this study were prepared in DMSO. It was found that some of the compounds showed significant antimicrobial effects (Table 4).

**Table 4:** Minimal inhibitory concentration (MIC) values of complexes against wild-type microorganisms (μg/ml).

Compound	Bacteria		Yeast
	*Enterococcus faecalis ATCC 51,299*	*Staphylococcus aureus ATCC 25,923*	*Candida albicans ATCC 10,231*
4a	> 6000	> 6000	> 6000
4b	750	750	> 6000
4c	> 6000	> 6000	> 6000
4d	3000	1500	> 6000
4e	> 6000	> 6000	> 6000
4f	> 6000	> 6000	> 6000
4g	> 6000	> 6000	> 6000
4h	375	> 6000	> 6000
6a	> 6000	> 6000	> 6000
6b	> 6000	> 6000	> 6000
6c	> 6000	> 6000	> 6000
6d	> 6000	> 6000	> 6000
6e	> 6000	> 6000	> 6000
6f	> 6000	> 6000	> 6000
6g	> 6000	> 6000	> 6000
6h	3000	750	> 6000
DMSO	> 6000	> 6000	> 6000
Gentamicin	> 256	4	ND
Flucanozole	ND	ND	32

When the MIC values of *E. faecalis* were examined, 4b and 4h showed significant antimicrobial activity. While the MIC value for 4b was 750 μg/mL, this value for 4h was found to be 375 μg/mL. As can be seen here, 4h has a stronger antimicrobial effect on *E. faecalis* than 4b. In addition, other compounds were found to have no antimicrobial effect on *E.faecalis*, but 4b and 4h had significant antimicrobial effect. MIC values were investigated for *S. aureus*, and it was found that these values ranged from 750 to > 6000 μg/ml. Among these compounds, mainly 4b and 4h were observed to have significant antimicrobial activity on *S. aureus*. The MIC values for 4b and 4h were determined to be 750 μg/mL. In addition to these results, no antimicrobial effect was observed for the MIC values of *C. albicans*.

Examination of the obtained results shows that 4b, 4h, and 6h, in particular, have a stronger antimicrobial effect than the other of the synthesized compounds. It was found that 4h showed more activity especially on *E. faecalis*. These results are due to the presence of nitro (NO_2_) group in the synthesized compound series unlike the others. In previous studies on this topic, compounds with similar structures were found to have significant antimicrobial activities^67,68^. In addition, compounds 4b and 6h have significant antimicrobial effects on *S. aureus*. In the literature, structures similar to these compounds have been shown to have significant antimicrobial effects on *S. aureus*^69,70^. *S. aureus* is an important bacterial pathogen in humans that can cause both community-acquired and nosocomial infections and can cause a variety of clinical manifestations including respiratory, urinary, skin, soft tissue, and bloodstream infections^71,72^. During the Covid 19 pandemic, some results have shown that *S. aureus* is associated with Covid 19 co-infection^73^. It is believed that the synthesized compounds can also be used for this purpose.

### Time-kill kinetics assay

**Figure 3:**
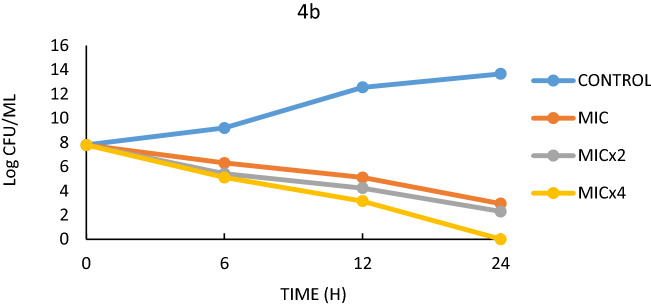
Effect of 4b on *E.* *faecalis.*

**Figure 4:**
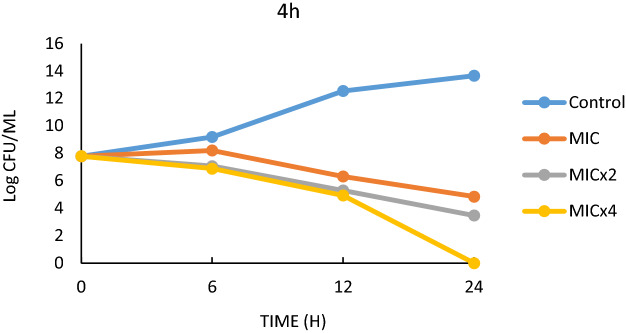
Effect of 4h on *E.* *faecalis*.

**Figure 5:**
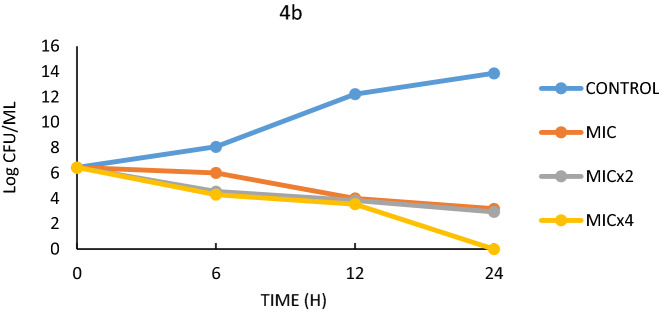
Effect of 4b on *S.* *aureus*.

**Figure 6:**
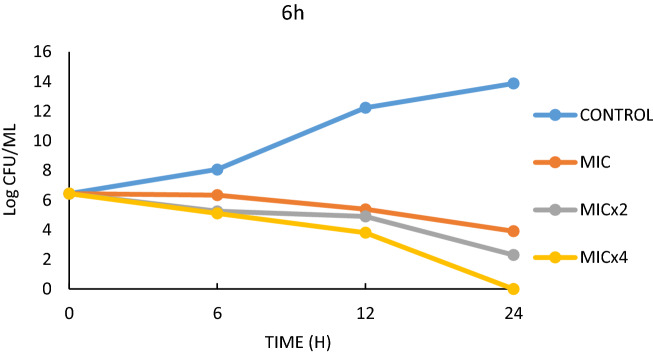
Effect of 6h on *S.* *aureus*.

In the time-killing study, compounds 4b, 4h, and 6h were realised to have the greatest antimicrobial activity. The effects of these compounds on *E. faecalis* and *S. aureus* were studied for 24 h. Figures 3, 4, 5, 6 and Table 5 show the growth curves and *t99* values of the bacteria as log cfu/mL. The *t99* value is expressed as the time equivalent to 2 log drops. In other words, *t99* refers to the time it takes for the bacterial count to decrease by 100 times the initial density.

**Table 5:** *t*_*99*_ values of the strains in the MIC, MICx2 and MICx4 concentrations.

Strain	Compounds		0.h (log)	24.h (log)	*T*_99_ (h)
*E.* *faecalis*	Control (*E.* *faecalis*)		7.79	13.66	> 24
	4b	MIC	7.79	2.95	10
		MICx2	7.79	2.30	9.09
		MICx4	7.79	0.00	7.14
	4h	MIC	7.79	4.85	16.6
		MICx2	7.79	3.47	11.1
		MICx4	7.79	0.00	7.14
*S.* *aureus*	Control (*S.* *aureus*)		6.44	13.87	> 24
	4b	MIC	6.44	3.18	10
		MICx2	6.44	2.94	9.52
		MICx4	6.44	0.00	6.89
	6h	MIC	6.44	3.90	20
		MICx2	6.44	2.30	11.76
		MICx4	6.44	0.00	6.89

Based on the MIC values of the antimicrobial results, three different MIC values (MIC, MICx2, MICx4) were determined in this study. Figures 3 and 4 show the life graph of *E. faecalis*. As can be seen here, it was found that the number of colonies of *E. faecalis* used as control group increased significantly within 24 h. In addition, it was found that when 4b was added to the medium, there was a significant decrease in the colony number of bacteria within 24 h for MIC, MICx2 and MICx4 values. When the t99 values were examined, it was found that the bacteria in the sample with MIC addition continued to live for 10 h. It was also found that the bacteria survived in the medium with MICx2 addition for 9.09 h and in the medium with MICx4 for 7.14 h. Thus, it was found that the increase in MIC values resulted in a significant decrease in bacterial longevity. Although it was observed that E.faecalis survived more than 24 h in the control sample (without 4h), the survival times were determined to be 16.6 h when 4h was added, 11.1 h when MICx2 was added, and 7.14 h when MICx4 was added. As can be seen here, increasing the MIC value has a significant effect on bacterial longevity.

Compounds 4b and 6h, which are thought to have potent antimicrobial activity on *S. aureus*, form another part of the time-killing study. In this study, bacteria were allowed to live in a nutrient medium for 24 h and colonies were counted at specific time periods. The colony count values were converted to *t99* values, and the survival times of the bacteria were determined. In the study conducted over 24 h, it was found that the number of colonies in the *S. aureus* control sample increased sharply. In the other experimental groups, 4b and 6h were added to the medium as MIC, MICx2 and MICx4, respectively (Figs. 5, 6). For the effect of 4b medium, it was found that *S.aureus* continued to live for 10 h for MIC value, 9.52 h for MICx2 value and 6.89 h for MICx4 value (Table 5). As can be seen here, increasing the MIC value has a negative effect on the bacteria's ability to form colonies and thus on their lifespan (Fig. 5). Serious differences in the colony forming ability of *S. aureus* were also observed in 6 h media with different MIC values. While the bacterium survived more than 24 h in the control sample, it was found that the bacterium survived less than 24 h in the samples to which 6 h was added at MIC, MICx2 and MICx4 values (Fig. 6). The *t99* values showed that the bacteria survived for 20 h at MIC value, 11.76 h at MICx2 value and 6.89 h at MICx4 value (Table 5).

In summary, it was found that the kill time values (*t99* value) for 4b were effective for approximately the same time for both bacteria at all concentrations. Consequently, the kinetics of kill time and antimicrobial activity results studied at different concentrations are mutually supportive. In fact, similar results were obtained in the study with *S. aureus*.

### Free radical scavenging activity

**Figure 7:**
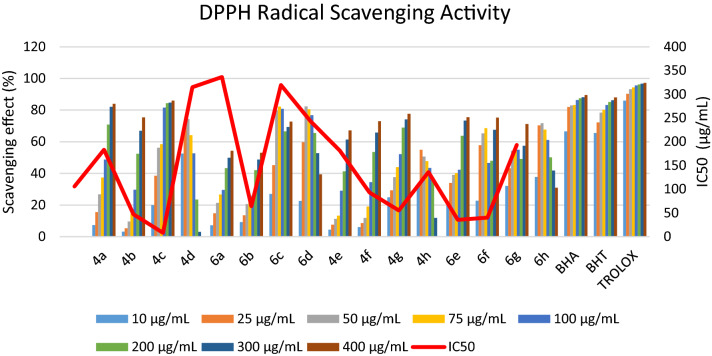
DPPH radical scavenging activity of synthesized compounds.

DPPH• (1,1-diphenyl-2-picryl-hydrazyl) is a stable free radical that accepts an electron or a hydrogen radical to become a stable diamagnetic molecule. The scavenging effect of the synthesized compounds (4a–h and 6a–h) and standards (BHA, BHT and Trolox) on the DPPH• radical is shown in Fig. 7.

The results indicate that the synthesized compounds have moderate to good free radical scavenging activity. Moreover, the radical scavenging activity increased with increasing concentration. Higher DPPH radical scavenging activity is associated with lower IC_50_ value.

### Docking study

In this study, molecular docking analysis was performed to evaluate the interaction and affinity of the synthesizing drug candidates towards three proteins: alanine racemase from *E. faecalis* (3E5P), biotin protein ligase from *S. aureus* (3V7R), COVID -19 major protease N3 complex (6LU7). Table 6, 7 and 8 show the binding energy and major residues to which the ligands bind. The compounds occupied the binding site of the target by hydrophobic interaction, hydrogen bonding, pi-cation interaction and pi-stacking. The hydrogen bonds of the derivatives are retained for most of our compounds, such as LYS40 for biotin-ptorein ligase of *S. aureus*, ARG227 for *E. faecalis*, and GLU166 for Mpro-Sars-Cov2. Compounds 4d, 4f, and 6h with the highest antibacterial activities exhibited strong affinity for the target enzyme with binding energy.

**Table 6:** The binding affinity of compounds 4a–h and 6a–h with *E. faecalis.*

Compound	Binding energy	Hydrophobic interaction	Hydrogen bond	π-cation interaction	π-stacking
4a	− 8.02	VAL38, LEU86, ASN206, TYR356	LYS40, TYR44, TYR356		
4b	− 8.48	ASN206, VAL225, TYR356	HIS169		
4c	− 7.91	VAL38, LEU86, ASN206, TYR356	LYS40, TYR44, VAL225, TYR356	LYS40,HIS169	
4d	− 7.85	LEU86, PHE167, ASN206, VAL225, TYR356	LYS40	LYS40,ARG139,HIS169	
4e	− 8.02	ASN206, TYR356	HIS169	LYS40	
4f	− 8.21	ASN206, TYR356	HIS169	LYS40	
4g	− 8.04	VAL38, ASN206	LYS40, ARG139, HIS169	LYS40,ARG139	HIS169
4h	− 8.79	VAL38, LEU86, ASN206, ARG222, TYR356	HIS169	LYS40	
6a	− 6.96	VAL38, LEU86, ASN206, VAL225	LYS40, TYR44, HIS169	LYS40	HIS169
6b	− 6.87	VAL38, TYR44, ASN206, ARG222, ILE354, TYR356	LYS40, ARG139, HIS169, ASN206, TYR356	LYS40	TYR356
6c	− 6.40	VAL38, ASN206, TYR356	LYS40, TYR44, VAL225, TYR356	ARG139	HIS169
6d	− 6.53	VAL38, ASN206, TYR356	LYS40, ARG139, HIS169, TYR356	LYS40	HIS169
6e	− 7.79	VAL38, LYS40, LEU86, ASN206, VAL225	LYS40, TYR44, HIS169		HIS169
6f	− 8.11	LEU86, ASN206	LYS40, TYR44, HIS169, TYR356		HIS169
6g	− 8.33	VAL38, LEU86, ASN206, VAL225, TYR356	LYS40, TYR44, ARG139, HIS169	LYS40	HIS169
6h	− 8.63	VAL38, LEU86, ASN206, VAL225, TYR356	LYS40, TYR44, TYR44, HIS169	LYS40,ARG139	HIS169

AlaR (PDB: 3E5P).

**Table 7:** The binding affinity of compounds 4a–h and 6a–h with *Staphylococcus aureus* biotin protein ligase (PDB: 3V7R).

Compound	Binding energy	Hydrophobic interaction	Hydrogen bonds	π-stacking	π-cation interactions
4a	− 7.59	ARG125, HIS126, TRP127, ILE224	LYS187, ARG227		
4b	− 7.65	ARG125, TRP127, LYS187, ILE224, ALA228	ARG125, LYS187, ARG227	HIS126	
4c	− 7.24		LYS187, ARG227		
4d	− 7.45	ARG125, TRP127, LYS187, PHE220, ILE224, ALA228	ARG125, ASP180, LYS187, ARG227	HIS126	
4e	− 8.28	ARG125, TRP127, LYS187, ILE224			
4f	− 8.26	ARG125, TRP127, LYS187, ILE224, ALA288	SER128, LYS187, ARG227		
4g	− 7.18	ARG125, HIS126, ILE224	ARG227		ARG125
4h	− 7.59	GLU115, ASP221			HIS126
6a	− 5.99	TRP127, LYS187, ILE224	HIS126, LYS187, ARG227		ARG227
6b	− 6.38	GLN116, SER129, SER130		HIS126	
6c	− 7.05	TRP127, LYS187, ILE224	HIS126, LYS187, ARG227		LYS187,ARG227
6d	− 6.11	ARG125, HIS126, TRP127, PHE220, ILE224, ALA228	SER128, LYS187, ARG227		
6e	− 6.73	TRP127, LYS187, ILE224	HIS126, ARG227		
6f.	− 6.32	ARG125, ILE224	HIS126, SER223	HIS126	
6g	− 6.17	HIS126, TRP127, LYS187, ILE224	HIS126, LYS187, ARG227		LYS187,ARG227
6h	− 8.78	TRP127, ILE224	SER223, ARG227

**Table 8:** The binding affinity of compounds 4a–h and 6a–h with Mpro-Sars-Cov2 (PDB: 6LU7).

Compound	Binding energy	Hydrophobic interaction	Hydrogen bond	π-stacking	π-cation interaction
4a	− 8.22	ASN142, MET165, GLN189	HIS163		
4b	− 8.61	ASP187,GLN189			
4c	− 7.67	MET165,GLN189	HIS163,GLU166		
4d	− 8.95	THR25,LEU27,HIS41,MET165,GLU166,GLN189	GLY143		
4e	− 8.39	HIS41, PHE140, MET165, GLU166, GLN189	GLU166	HIS163	
4f	− 9.04	HIS41, PHE140, MET165, GLU166, GLN189	GLU166	HIS163	
4g	− 7.65	THR25, LEU27, MET165, GLN189	GLY143, GLU166, GLN189	HIS41	HIS41
4h	− 7.62	MET165, ASP187		HIS41	
6a	− 7.76	MET165, LEU167, GLN192, PRO168	GLU166		
6b	− 8.22	MET165, GLU166, GLN189	HIS41		
6c	− 7.78	MET165,GLN189, LEU167, GLN192	GLU166		
6d	− 8.81	MET165,GLN189, LEU167, GLN192, PRO168	GLU166		
6e	− 7.5	MET165, GLU166, PRO168, GLN189	GLU166, GLU189		
6f	− 7.63	MET165, GLU166, PRO168, GLN189, ALA191			
6g	− 6.13	MET165, PRO168, GLN189	GLU166, GLU189		
6h	− 6.37	ASN180, PRO184, VAL186	PHE181, PHE185		

**Figure 8:**
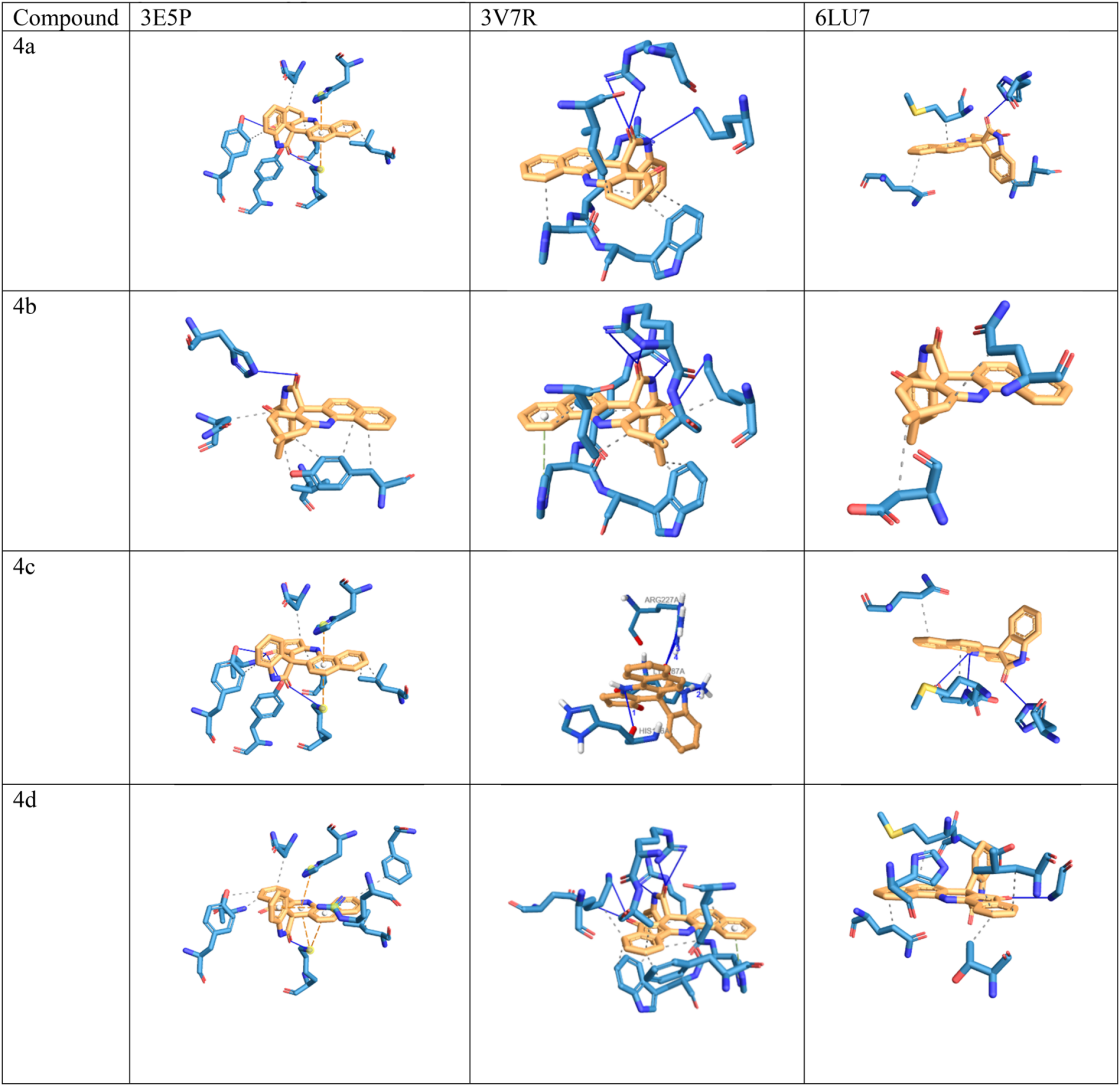

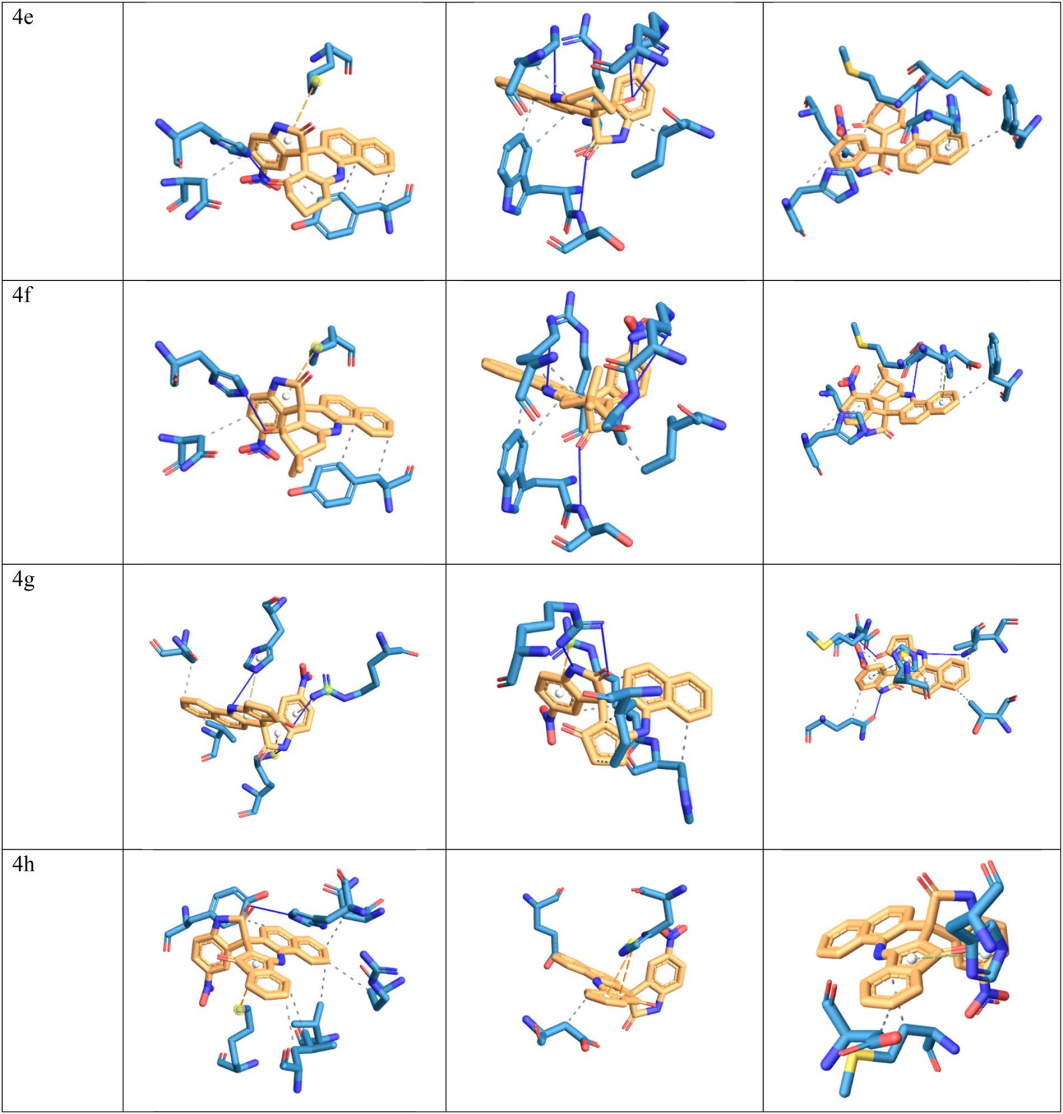

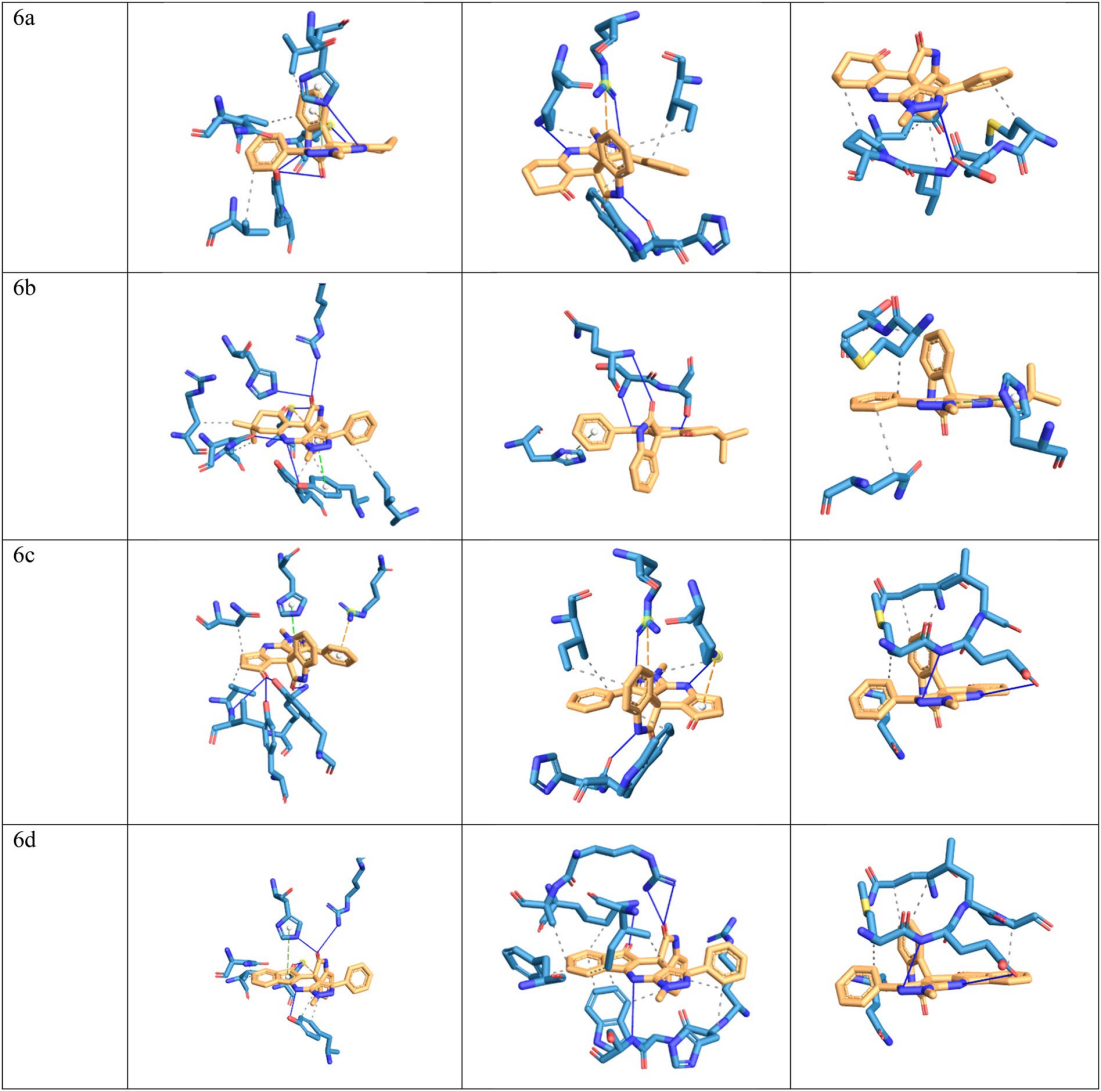

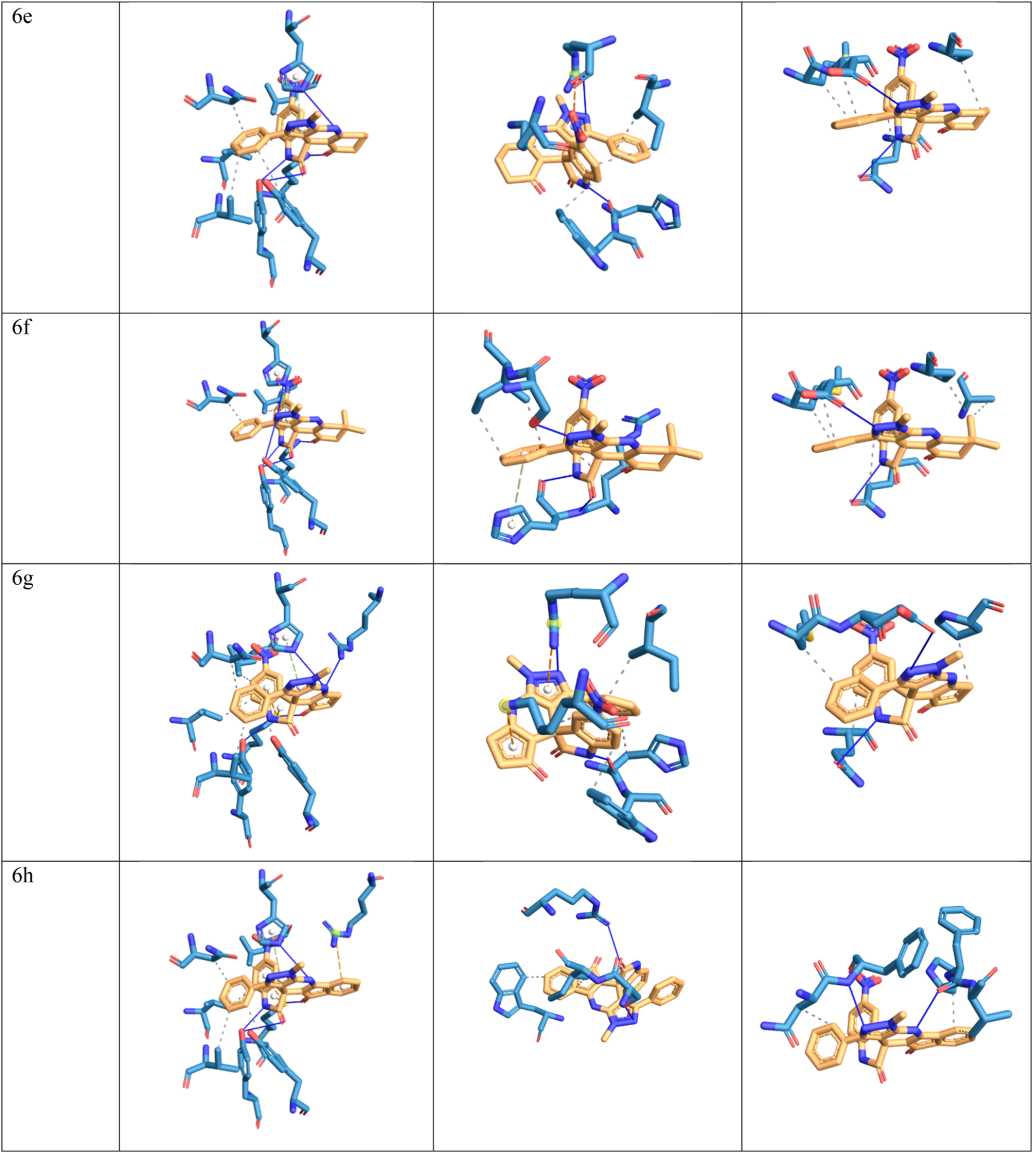
Docking possition of compounds 4a–h, 6a–h in the active site of 3E5P, 3V7R, 6LU7.

Meanwhile compound 6h and 4b was docked with *S.aureus* protein exhibited the highest binding energy of − 8.78 and − 7.65 kcal/mol respectively. Furthermore hydrogen bond was formed with HIS169 for protein 3E5P, ARG227 for 3V7R, and GLU166 for 6LU7 as illustrated in Fig. 8.

Moreover, compounds 4b, 4h, and 6h had the highest antimicrobial activty and also revealed good binding energies were − 8.79, − 8.63, − 8.48 kcal/mol.

Compounds 6h and 4b, which were docked to the *S. aureus* protein, had the highest binding energy of − 8.78 and 7.65 kcal/mol, respectively. In addition, hydrogen bonds were formed with HIS169 for protein 3E5P, ARG227 for 3V7R, and GLU166 for 6LU7, as shown in Fig. 8 Moreover, compounds 4b, 4h and 6h exhibited the highest antimicrobial activity and also showed good binding energies of − 8.79, − 8.63, − 8.48 kcal/mol, respectively.

## Conclusions

In conclusion, spiro[benzo[*h*]quinoline-7,3′-indoline]dione derivatives and spiro[indoline-3,4′-pyrazolo[3,4-*b*]quinoline]dione derivatives were synthesized via one-pot three-component reactions of isatins, naphthalene-1-amine or 5-amino-1-methyl-3-pheylpyrazole, and 1,3-dicarbonyl compounds in the presence of ( ±)-camphor-10-sulfonic acid in H_2_O/EtOH solvent under ultrasound-promoted conditions. This procedure represents a useful, simple and effective green approach to obtain spiro[benzo[*h*]quinoline-7,3′-indoline]diones and spiro[indoline-3,4′-pyrazolo[3,4-*b*]quinoline]diones in high yields. The synthesized compounds showed moderate to good free radical scavenging activities.

From the present studies, isatin-based quinoline-pyrazole-indoline hybrids have shown good antimicrobial and antioxidant activities and effects on covid-19.

The antimicrobial activities of these compounds were determined. Compounds 4b, 4h and 6h showed higher activities.

Compound 4b showed promising broad spectrum antibacterial activities against *S. aureus* and *E. faecalis*, which could be attributed to the incorporated alkylated cyclohexanone moiety of spirooxindole.

On the other hand, compound 4h showed significant antibacterial activity against *Enterococcus faecalis* (MIC of 375 µg/mL).

Docking studies for these compounds were performed to gain insight into the mode of action, indicating that the best binding with isatin moiety was all compounds with hydrogen bonds formed by the Lys40, His169 for inhibition of 3E5P, Arg227, His126 for inhibition of 3V7R and GLU166 for inhibition of 6LU7.

Moreover, compounds 4h, 6h, and 4b had the highest antimicrobial activity and also showed good binding energies of − 8.79, − 8.63, − 8.48 kcal/mol, respectively. However, other experimental studies on covid activity may also support docking studies.

## Experimental

### Materials and methods

The NMR spectra were recorded on a Bruker Avance III-500 MHz NMR. Chemical shifts are given in ppm downfield from Me_4_Si in DMSO-d_6_ solution. Coupling constants are given in Hz. MS spectra were performed on an AB Sciex 3200 QTRAP LC-MS/MS**.** The FTIR spectra were recorded on a Perkin-Elmer FT-IR spectrometer (ATR) and absorption frequencies are reported in cm^−1^. Elemental analyses were performed with CHNS-932 LECO apparatus and were in good agreement (± 0.2%) with the calculated values. Ultrasonication was performed in an Alex Ultrasonic Bath with a frequency of 32 kHz and a power of 230 W. Melting points were measured on a Gallenkamp melting-point apparatus. TLC was performed on standard conversion aluminum sheets pre-coated with a 0.2-mm layer of silica gel. All of the reagents were commercially available.

### General procedure for the synthesis of compounds (4a–h and 6a–h) under ultrasonic irradiation

CSA (0.05 mmol) was added to a solution of 5-amino-1-methyl-3-phenylpyrazole (1.00 mmol) or 1-naphthylamine (1.00 mmol), isatin (1.00 mmol), and β-diketones (1.00 mmol) in H_2_O/EtOH (3/1, v/v, 8 mL) at room temperature and then the reaction mixture was sonicated at 50 °C for the time indicated in Tables 2, 3. After completion of the reaction, as indicated by TLC monitoring, the resultant solid was washed with water and ethanol and then recrystallized from ethanol to give products 4a–h and 6a–h.

### 10,11-dihydro-8*H*-spiro[benzo[*c*]acridine-7,3′-indoline]-2′,8(9*H*,12*H*)-dione (4a)

White powder, 0.322 g (88%). Mp: 278–281 °C. FTIR (ATR): v = 3362 (N–H), 3295 (N–H), 3059 (aromatic = C–H), 2939 (aliphatic C–H), 1697 (C=O), 1604, 1514 (aromatic C=C), 1499, 1469 (aliphatic C–H), 1333, 1275 (aliphatic C–C), 1086 (C–N) cm^-1^. ^1^H NMR (d_6_-DMSO, 500 MHz): δ = 1.85–1.96 (m, 2H, aliphatic CH_2_), 2.12–2.21 (m, 2H, aliphatic CH_2_), 2.79–2.88 (m, 2H, aliphatic CH_2_), 6.39–7.79 (m, 8H, Ar H), 8.33 (d, *J* = 8.34, 1H, Ar H), 8.50 (d, *J* = 8.49 Hz, 1H, Ar–H), 9.88 (s, 1H, NH), 10.39 (s, 1H, NH) ppm. ^13^C NMR (d_6_-DMSO, 126 MHz): δ = 21.09 (aliphatic CH_2_), 27.21 (aliphatic CH_2_), 36.71 (aliphatic CH_2_), 51.65 (quarternary C), 106.95 (Ar C), 107.21(Ar C), 108.85 (Ar C), 118.52 (Ar C), 121.39 (Ar C), 121.48 (Ar C), 122.11 (Ar C), 123.08 (Ar C), 123.36 (Ar C), 124.15 (Ar C), 126.30 (Ar C), 127.29 (Ar C), 128.02 (Ar C), 129.96 (Ar C), 132.50 (Ar C), 140.33 (Ar C), 141.16 (Ar C), 154.51 (Ar C), 180.98 (C=O), 192.83 (C=O) ppm. MS: *m/z* (ESI): 366, 367; Anal. Calcd for C_24_H_18_N_2_O_2_: C, 78.67; H, 4.95; N, 7.65; O, 8.73; Found: C, 78.91; H, 4.78; N, 7.86%.

### 10,10-dimethyl-10,11-dihydro-8*H*-spiro[benzo[*c*]acridine-7,3′-indoline]-2',8(9*H*,12*H*)-dione (4b)^44^

White powder, 0.330 g (84%). Mp: 284–286 °C. FTIR (ATR): v = 3516 (N–H), 3252 (N–H), 3008 (aromatic = C–H), 2957 (aliphatic C-H), 1739, 1721 (C=O), 1609, 1592 (aromatic C=C), 1485, 1461 (aliphatic C–H), 1333, 1266 (aliphatic C–C), 1133, 1093 (C–N) cm^−1^. ^1^H NMR (d_6_-DMSO, 500 MHz): δ = 1.04 (s, 3H, aliphatic CH_3_), 1.07 (s, 3H, aliphatic CH_3_), 2.00 (d, *J* = 16.0 Hz, 1H, aliphatic CH_2_), 2.17 (br s, 1H, aliphatic CH_2_), 2.51 (m, 2H, aliphatic CH_2_), 6.68 (d, *J* = 8.7 Hz, 1H, Ar H), 6.75–6.92 (m, 3H, Ar H), 7.09 (t, *J* = 8.2 Hz, 1H, Ar H), 7.38 (m, 1H, Ar H), 7.53 (t, *J* = 7.4 Hz, 1H, Ar H), 7.61 (t, *J* = 7.6 Hz, 1H, Ar H), 7.79 (d, *J* = 8.1 Hz, 1H, Ar H), 8.49 (d, *J* = 8.6 Hz, 1H, Ar H), 9.35 (s, 1H, NH), 10.40 (s, 1H, NH) ppm. ^13^C NMR (d_6_-DMSO, 126 MHz): δ = 18.50 (aliphatic CH_3_), 27.01 (aliphatic CH_3_), 28.45(aliphatic C(CH_3_)_2_), 32.15 (aliphatic CH_2_), 50.31 (aliphatic CH_2_), 56.01 (quarternary C), 105.94 (Ar C), 108.93 (Ar C), 118.62 (Ar C), 121.39 (Ar C), 122.17 (Ar C), 123.06 (Ar C), 123.19 (Ar C), 124.14 (Ar C), 124.18 (Ar C), 126.02 (Ar C), 126.26 (Ar C), 127.27 (Ar C), 127.99 (Ar C), 130.20 (Ar C), 132.56 (Ar C), 140. 19 (Ar C), 141.26 (Ar C), 152. 56 (Ar C), 180.82 (C=O), 192.48 (C=O) ppm. MS: *m/z* (ESI): 394, 395; Anal. Calcd for C_26_H_22_N_2_O_2_: C, 79.16; H, 5.62; N, 7.10; O, 8.11; Found: C, 78.98; H, 5.49; N, 7.27%.

### 9,10-dihydrospiro[benzo[*h*]cyclopenta[*b*]quinoline-7,3'-indoline]-2′,8(11*H*)-dione (4c)

White powder, 0.299 g (85%). Mp: 281–283 °C. FTIR (ATR): v = 3219 (N–H), 3040 (aromatic = C–H), 2955 (aliphatic C–H), 1712 (C=O), 1607, 1515 (aromatic C=C), 1463, 1381 (aliphatic C–H), 1333, 1228 (aliphatic C=C), 1093, 1064 (C-N) cm^−1^. ^1^H NMR (d_6_-DMSO, 500 MHz): δ = 2.29–2.32 (m, 2H, aliphatic CH_2_), 2.88–2.92 (m, 2H, aliphatic CH_2_), 6.85–6.95 (m, 2H, Ar H), 7.12–7.19 (m, 3H, Ar H), 7.41–7.64 (m, 5H), 10.30 (s, 1H, NH), 10.46 (s, 1H) ppm. ^13^C NMR (d_6_-DMSO, 126 MHz): δ = 24.87 (aliphatic CH_2_), 33.02 (aliphatic CH_2_), 56.02 (quarternary C), 109.22 (Ar C), 110.44 (Ar C), 117.49 (Ar C), 118.42 (Ar C), 121.26 (Ar C), 121.96 (Ar C), 122.69 (Ar C), 123.46 (Ar C), 124.38 (Ar C), 124.84 (Ar C), 126.24 (Ar C), 126.44 (Ar C), 128.09 (Ar C), 131.79 (Ar C), 132.82 (Ar C), 138.24 (Ar C), 141.43 (Ar C), 166.13 (Ar C), 179.38 (C = O), 199.06 (C = O) ppm. MS: *m/z* (ESI): 352, 353; Anal. Calcd for C_23_H_16_N_2_O_2_: C, 78.39; H, 4.58; N, 7.95; O, 9.08; Found: C, 78.53; H, 4.46; N, 8.09%.

### Spiro[benzo[*h*]indeno[1,2-*b*]quinoline-7,3'-indoline]-2',8(13*H*)-dione (4d)^44^

Red powder, 0.356 g (89%). Mp: 297–299 °C. FTIR (ATR): v = 3345 (N–H), 3086 (aromatic = C–H), 2912 (aliphatic C–H), 1702 (C=O), 1621, 1590 (aromatic C=C), 1483, 1468 (aliphatic C–H), 1350, 1278 (aliphatic C–C), 1119, 1089 (C–N) cm^-1^. ^1^H NMR (d_6_-DMSO, 500 MHz): δ = 6.62–6.79 (m, 3H, Ar H), 6.90–7.12 (m, 4H, Ar H), 7.18–7.70 (m, 4H, Ar H), 7.90–8.70 (m, 3H, Ar H), 10.38 (s, 1H, NH), 10.63 (s, 1H, NH) ppm. ^13^C NMR (d_6_-DMSO, 126 MHz): δ = 52.41 (quarternary C), 109.52 (Ar C), 109.85 (Ar C), 120.09 (Ar C), 120.61 (Ar C), 121.49 (Ar C), 122.18 (Ar C), 123.42 (Ar C), 124.58 (Ar C), 126.51 (Ar C), 126.76 (Ar C), 128.19 (Ar C), 128.30 (Ar C), 128.79 (Ar C), 130.40 (Ar C), 131.36 (Ar C), 132.86 (Ar C), 133.74 (Ar C), 135.88 (Ar C), 136.32 (Ar C), 137.96 (Ar C), 141.41 (Ar C), 142.05 (Ar C), 142.70 (Ar C), 156.02 (Ar C), 175.68 (C = O), 189.15 (C = O) ppm. MS: *m/z* (ESI): 400, 401; Anal. Calcd for C_27_H_16_N_2_O_2_: C, 80.99; H, 4.03; N, 7.00; O, 7.99; Found: C, 81.17; H, 4.18; N, 6.88%.

### 5′-nitro-10,11-dihydro-8*H*-spiro[benzo[*c*]acridine-7,3'-indoline]-2′,8(9*H*,12*H*)-dione (4e)

White powder, 0.349 g (85%). Mp: 287–290 °C. FTIR (ATR): v = 3502 (N–H), 3298 (N–H), 3076 (aromatic = C–H), 2946 (aliphatic C–H), 1707 (C=O), 1653, 1621 (aromatic C=C), 1511, 1479 (aliphatic C–H), 1331, 1280 (aliphatic C–C), 1079 (C–N) cm^-1^. ^1^H NMR (d_6_-DMSO, 500 MHz): δ = 1.83–1.96 (m, 2H, aliphatic CH_2_), 2.15–2.24 (m, 2H, aliphatic CH_2_), 2.76–2.92 (m, 2H, aliphatic CH_2_), 6.56–7.65 (m, 7H, Ar H), 8.13–8.54 (m, 2H, Ar H), 10.33 (s, 1H, NH), 11.16 (s, 1H, NH) ppm.^13^C NMR (d_6_-DMSO, 126 MHz): δ = 21.45 (aliphatic CH_2_), 27.63 (aliphatic CH_2_), 36.90 (aliphatic CH_2_), 52.22 (quarternary C), 106.76 (Ar C), 109.63 (Ar C), 117.38 (Ar C), 119.12 (Ar C), 122.04 (Ar C), 122.67 (Ar C), 123.56 (Ar C), 124.05 (Ar C), 125.56 (Ar C), 126.83 (Ar C), 127.16 (Ar C), 128.59 (Ar C), 130.71 (Ar C), 133.19 (Ar C), 141.16 (Ar C), 142.65 (Ar C), 148.55 (Ar C), 155.92 (Ar C), 181.78 (C=O), 193.71 (C=O) ppm. MS: *m/z* (ESI): 411, 412; Anal. Calcd for C_24_H_17_N_3_O_4_: C, 70.07; H, 4.16; N, 10.21; O, 15.56; Found: C, 70.23; H, 4.28; N, 10.13%.

### 10,10-dimethyl-5′-nitro-10,11-dihydro-8*H*-spiro[benzo[*c*]acridine-7,3′-indoline]-2′,8(9*H*,12*H*)-dione (4f)

White powder, 0.381 g (87%). Mp: 292–295 °C. FTIR (ATR): v = 3504 (N–H), 3319 (N–H), 3080 (aromatic = C-H), 2956 (aliphatic C-H), 1713 (C = O), 1652, 1625 (aromatic C = C), 1483, 1412 (aliphatic C-H), 1381, 1250 (aliphatic C–C), 1176, 1075 (C-N) cm^-1^. ^1^H NMR (d_6_-DMSO, 500 MHz): δ = 1.05 (s, 3H, aliphatic CH_3_), 1.08 (s, 3H, aliphatic CH_3_), 1.99–2.21 (m, 2H, aliphatic CH_2_), 2.66–2.75 (m, 2H, aliphatic CH_2_), 6.58–6.65 (m, 3H, Ar H), 7.07–7.56 (m, 3H, Ar H), 7.62–8.50 (m, 3H, Ar H), 10.70 (s, 1H, NH), 11.17 (s, 1H, NH) ppm. ^13^C NMR (d_6_-DMSO, 126 MHz): δ = 27.82 (aliphatic CH_3_), 28.45 (aliphatic CH_3_), 32.79 (aliphatic C(CH_3_)_2_), 50.36 (aliphatic CH_2_), 52.14 (CH_2_), 56.47 (quarternary C), 105.39 (Ar C), 109.73 (Ar C), 117.35 (Ar C), 118.85 (Ar C), 122.03 (Ar C), 122.69 (Ar C), 124.05 (Ar C), 124.21 (Ar C), 125.60 (Ar C), 126.88 (Ar C), 127.18 (Ar C),128.60 (Ar C), 130.85 (Ar C), 133.21 (Ar C), 141.03 (Ar C), 142.60 (Ar C), 148.61 (Ar C), 154.01 (Ar C), 181.69 (C = O), 193.44 (C = O) ppm. MS: *m/z* (ESI): 439, 440; Anal. Calcd for C_26_H_21_N_3_O_4_: C, 71.06; H, 4.82; N, 9.56; O, 14.56; Found: C, 70.91; H, 4.94; N, 9.47%.

### 5′-nitro-9,10-dihydrospiro[benzo[*h*]cyclopenta[*b*]quinoline-7,3′-indoline]-2′,8(11*H*)-dione (4g)

White powder, 0.357 g (90%). Mp: > 300 °C. FTIR (ATR): v = 3208 (N–H), 3032 (aromatic C–H), 2962 (aliphatic C–H), 1718 (C=O), 1605, 1510 (aromatic C=C), 1460, 1380 (aliphatic C–H), 1330, 1226 (aliphatic C–C), 1080, 1065 (C–N) cm^-1^. ^1^H NMR (d_6_-DMSO, 500 MHz): δ = 2.26–2.30 (m, 2H, aliphatic CH_2_), 2.79–2.97 (m, 2H, aliphatic CH_2_), 6.58–6.60 (m, 1H, Ar H), 7.12–7.15 (m, 1H, ArH), 7.43–7.45(m, 2H, ArH), 7.64–7.69 (m, 2H, Ar H), 7.83–8.16 (m, 3H, Ar H), 10.52 (s, 1H, NH), 11.28 (s, 1H, NH) ppm.^13^C NMR (d_6_-DMSO, 126 MHz): δ = 25.53 (aliphatic CH_2_), 33.39 (aliphatic CH_2_), 51.75 (quarternary C), 109.97 (Ar C), 110.16 (Ar C), 117.20 (Ar C), 120.30 (Ar C), 121.87 (Ar C), 122.62 (Ar C), 123.19 (Ar C), 124.24 (Ar C), 124.93 (Ar C), 126.07 (Ar C), 127.03 (Ar C), 128.69 (Ar C), 132.56 (Ar C), 133.45 (Ar C), 139.03 (Ar C), 142.97 (Ar C), 148.63 (Ar C), 167.24 (Ar C), 180.27 (C = O), 199.87 (C = O) ppm. MS: *m/z* (ESI): 397, 398; Anal. Calcd for C_23_H_15_N_3_O_4_: C, 69.52; H, 3.80; N, 10.57; O, 16.10; Found: C, 69.68; H, 3.73; N, 10.37%.

### 5′-nitrospiro[benzo[*h*]indeno[1,2-*b*]quinoline-7,3'-indoline]-2′,8(13*H*)-dione (4h)^44^

Red powder, 0.409 g (92%). Mp: < 300 °C. FTIR (ATR): v = 3336 (N–H), 3082 (aromatic C-H), 2918 (aliphatic C-H), 1705 (C=O), 1618, 1586 (aromatic C=C), 1480, 1460 (aliphatic), 1352, 1275 (aliphatic C–C), 1117, 1080 (C–N) cm^-1^. ^1^H NMR (d_6_-DMSO, 500 MHz): δ = 6.62–6.97 (m, 3H, Ar H), 7.19–7.43 (m, 3H, Ar H), 7.53–7.62 (m, 3H, Ar H), 7.93–8.55 (m, 4H, Ar H), 10.51 (s, 1H, NH), 11.40 (s, 1H, NH) ppm. ^13^C NMR (d_6_-DMSO, 126 MHz): δ = 52.11 (quaternary C, C-11), 110.44 (Ar C, C-12), 110.61 (Ar C, C-30), 118.82 (Ar C, C-7), 120.62 (Ar C, C-9), 120.77 (Ar C, C-6), 121.53 (Ar C, C-29), 122.52 (Ar C, C-21), 123.33 (Ar C, C-5), 125.04 (Ar C, C-1), 125.64 (Ar C, C-8), 126.32 (Ar C, C-2), 126.89 (Ar C, C-27), 127.54 (Ar C, C-18), 128.74 (Ar C, C-3), 129.72 (Ar C, C-19), 132.12 (Ar C, C-4), 133.54 (Ar C, C-20), 136.70 (Ar C, C-17), 136.92 (Ar C, C-23), 138.80 (Ar C, C-16), 142.08 (Ar C, C-10), 143.20 (Ar C-28), 149.94 (Ar C-24), 156.95 (Ar C, C-13), 176.93 (C=O, C-26), 180.39 (C = O, C-15) ppm. MS: *m/z* (ESI): 445, 446; Anal. Calcd for C_27_H_15_N_3_O_4_: C, 72.80; H, 3.39; N, 9.43; O, 14.37; Found: C, 72.69; H, 3.62; N, 9.30%.

### 1′-methyl-3′-phenyl-6′,7′,8′,9′-tetrahydrospiro[indoline-3,4′-pyrazolo[3,4-*b*]quinoline]-2,5′(1′*H*)-dione (6a)

White powder, 0.332 g (84%). Mp: 276–279 °C. FTIR (ATR): v = 3356 (N–H), 3238 (N–H), 3006 (aromatic = C–H), 2937 (aliphatic C–H), 1690 (C=O), 1620, 1546 (aromatic C=C), 1493, 1466 (aliphatic C–H), 1324, 1254 (aliphatic C–C), 1135, 1086 (C–N) cm^-1^. ^1^H NMR (d_6_-DMSO, 500 MHz): δ = 1.81–1.93 (m, 2H, aliphatic CH_2_), 2.06–2.17 (m, 2H, aliphatic CH_2_), 2.69 (d, *J* = 4.3 Hz, 2H, aliphatic CH_2_), 3.77 (s, 3H, N–CH_3_), 6.45 (d, *J* = 7.5 Hz, 1H, Ar H), 6.53 (d, *J* = 7.3 Hz, 2H, Ar H), 6.80 (t, *J* = 7.2 Hz, 1H, Ar H), 6.88 (d, *J* = 7.0 Hz, 1H, Ar H), 6.98–7.08 (m, 3H, Ar H), 7.18 (t, *J* = 7.2 Hz, 1H, Ar H), 9.70 (s, 1H, NH), 10.04 (s, 1H, NH) ppm. ^13^C NMR (d_6_-DMSO, 126 MHz): δ = 21.01 (aliphatic CH_2_), 27.72 (aliphatic CH_2_), 35.06 (aliphatic CH_2_), 37.12 (N-CH_3_), 50.12 (quarternary C), 99.81 (Ar C), 108.52 (Ar C), 108.92 (Ar C), 120.90 (Ar C), 123.03 (Ar C), 127.01 (Ar C), 127.06 (Ar C), 127.28 (Ar C), 128.39 (Ar C), 129.12 (Ar C), 132.50 (Ar C), 133.39 (Ar C), 137.23 (Ar C), 138.55 (Ar C), 142.30 (Ar C), 147.02 (Ar C), 153.88 (Ar C), 179.66 (C=O), 193.12 (C=O) ppm. MS: *m/z* (ESI): 396, 397; Anal. Calcd for C_24_H_20_N_4_O_2_: C, 72.71; H, 5.08; N, 14.13; O, 8.07; Found: C, 72.30; H, 5.15; N, 13.98%.

### 1′,7′,7′-trimethyl-3′-phenyl-6′,7′,8′,9′-tetrahydrospiro[indoline-3,4′-pyrazolo[3,4-*b*]quinoline]-2,5′(1′*H*)-dione (6b)

Beige powder, 0.368 g (87%). Mp: 278–281 °C. FTIR (ATR): v = 3618 (N–H), 3320 (N–H), 3087, 3011 (aromatic = C–H), 2988, 2956 (aliphatic C–H), 1682 (C=O), 1629, 1576 (aromatic C=C), 1472, 1406 (aliphatic C–H), 1366, 1320, 1285 (aliphatic C–C), 1196, 1084 (C–N) cm^-1^. ^1^H NMR (d_6_-DMSO, 500 MHz): δ = 1.01 (s, 3H, aliphatic CH_3_), 1.04 (s, 3H, aliphatic CH_3_), 1.96 (d, *J* = 16.0 Hz, 1H, aliphatic CH_2_), 2.08 (d, *J* = 16.1 Hz, 1H, aliphatic CH_2_), 2.51 (dd, *J*_*1*_ = 3.5 Hz, *J*_*2*_ = 1.8 Hz, 2H, aliphatic CH_2_), 3.78 (s, 3H, N–CH_3_), 6.47 (d, *J* = 7.6 Hz, 1H, Ar H), 6.54–6.57 (m, 2H, Ar H), 6.79–6.83 (m, 1H, Ar H), 6.88 (m, 1H, Ar H), 7.01–7.07 (m, 3H, Ar H), 7.16–7.20 (m, 1H, Ar H), 9.72 (s, 1H, NH), 10.01 (s, 1H, NH) ppm. ^13^C NMR (d_6_-DMSO, 126 MHz): δ = 26.82 (aliphatic CH_3_), 28.13 (aliphatic CH_3_), 32.04 (aliphatic C(CH_3_)_2_), 35.07 (N-CH_3_), 41.00 (aliphatic CH_2_), 49.01 (aliphatic CH_2_), 50.56 (quarternary C), 99.81 (Ar C), 107.64 (Ar C), 108.59 (Ar C), 120.93 (Ar C), 122.91 (Ar C), 123.06 (Ar C), 127.08 (Ar C), 127.29 (Ar C), 128.37 (Ar C), 133.41 (Ar C), 137.37 (Ar C), 138.47 (Ar C), 142.35 (Ar C), 147.02 (Ar C), 151.92 (Ar C), 179.57 (C=O), 192.82 (C=O) ppm. MS: *m/z* (ESI): 424, 425; Anal. Calcd for C_26_H_24_N_4_O_2_: C, 73.56; H, 5.70; N, 13.20; O, 7.54; Found: C, 73.62; H, 5.53; N, 13.43%.

### 1-methyl-3-phenyl-6,7-dihydro-1*H*-spiro[cyclopenta[*e*]pyrazolo[3,4-*b*]pyridine-4,3′-indoline]-2′,5(8*H*)-dione (6c)

White powder, 0.343 g (90%). Mp: > 300 °C. FTIR (ATR): v = 3485 (N–H), 3315 (N–H), 3119, 3007 (aromatic = C–H), 2925, 2815 (aliphatic C–H), 1666 (C=O), 1612, 1587 (aromatic C=C), 1487, 1470 (aliphatic C–H), 1357, 1290 (aliphatic C–C gerilmeleri), 1177, 1074 (C–N) cm^-1^. ^1^H NMR (d_6_-DMSO, 500 MHz): δ = 2.26 (dd, *J*_*1*_ = 10.8 Hz, *J*_*2*_ = 5.0 Hz, 2H, aliphatic CH_2_), 2.71–2.80 (m, 2H, aliphatic CH_2_), 3.81 (s, 3H, N–CH_3_), 6.61 (d, *J* = 7.7 Hz, 1H, Ar H), 6.68–6.72 (m, 2H, Ar H), 6.84 (t, *J* = 7.4 Hz, 1H, Ar H), 6.90 (d, *J* = 7.1 Hz, 1H, Ar H), 7.04 (t, *J* = 7.7 Hz, 2H, Ar H), 7.09 (td, *J*_*1*_ = 7.6 Hz, *J*_*2*_ = 1.1 Hz, 1H, Ar H), 7.16 (t, *J* = 7.4 Hz, 1H, Ar H) 10.13 (s, 1H, NH), 10.83 (s, 1H, NH) ppm. ^13^C NMR (d_6_-DMSO, 126 MHz): δ = 23.95 (aliphatic CH_2_), 33.42 (aliphatic CH_2_), 35.15 (N–CH_3_), 47.74 (quarternary C), 99.58 (Ar C), 108.87 (Ar C), 113.10 (Ar C), 121.44 (Ar C), 123.95 (Ar C), 127.38 (Ar C), 127.70 (Ar C), 128.04 (Ar C), 131.30 (Ar C), 136.47 (Ar C), 139.77 (Ar C), 142.00 (Ar C), 147.15 (Ar C), 166.13 (Ar C), 178.34 (C=O), 198.81 (C=O) ppm. MS: *m/z* (ESI): 382, 383; Anal. Calcd for C_23_H_18_N_4_O_2_: C, 72.24; H, 4.74; N, 14.65; O, 8.37; Found: C, 72.38; H, 4.53; N, 14.59%.

### 1-methyl-3-phenyl-1*H*-spiro[indeno[2,1-*e*]pyrazolo[3,4-*b*]pyridine-4,3′-indoline]-2′,5(10*H*)-dione (6d)

Red powder, 0.382 g (89%). Mp: > 300 °C. FTIR (ATR): v = 3357 (N–H), 3046, 3020 (aromatic = C–H), 2914 (aliphatic C–H), 1684 (C=O), 1664, 1620 (aromatic C=C), 1483, 1470 (aliphatic C–H), 1340, 1255 (aliphatic C–C), 1070 (C–N) cm^−1^. ^1^H NMR (d_6_-DMSO, 500 MHz): δ = 3.96 (s, 3H, N–CH_3_), 6.67 (d, *J* = 7.3 Hz, 1H, Ar H), 6.78 (m, 2H, Ar H), 6.84 (t, *J* = 7.3 Hz, 1H, ArH), 6.99 (d, *J* = 7.0 Hz, 1H, Ar H), 7.02–7.15 (m, 3H, Ar H), 7.18 (t, *J* = 7.6 Hz, 2H, Ar H), 7.37 (t, *J* = 6.7 Hz, 1H, Ar H), 7.50 (d, *J* = 6.6 Hz, 1H, Ar H), 7.78 (d, *J* = 6.7 Hz, 1H, Ar H), 10.18 (s, 1H, NH), 11.04 (s, 1H, NH) ppm. ^13^C NMR (d_6_-DMSO, 126 MHz): δ = 35.81 (N–CH_3_), 47.46 (quarternary C), 101.05 (Ar C), 106.00 (Ar C), 109.09 (Ar C), 119.65 (Ar C), 120.22 (Ar C), 121.57 (Ar C), 123.09 (Ar C), 123.87 (Ar C), 124.05 (Ar C), 127.16 (Ar C), 127.40 (Ar C), 127.79 (Ar C), 127.94 (Ar C), 130.35 (Ar C), 131.64 (Ar C), 133.15 (Ar C), 133.89 (Ar C), 136.08 (Ar C), 136.36 (Ar C), 139.27 (Ar C), 141.95 (Ar C), 147.50 (Ar C), 155.67 (Ar C), 178.44 (C=O), 188.67 (C=O) ppm. MS: *m/z* (ESI): 430, 431; Anal. Calcd for C_27_H_18_N_4_O_2_: C, 75.34; H, 4.21; N, 13.02; O, 7.43; Found: C, 75.28; H, 4.11; N, 13.26%.

### 1′-methyl-5-nitro-3′-phenyl-6′,7′,8′,9′-tetrahydrospiro[indoline-3,4′-pyrazolo[3,4-*b*]quinoline]-2,5′(1′*H*)-dione (6e)

White powder, 0.379 g (86%). Mp: 283–286 °C. FTIR (ATR): v = 3632 (N–H), 3222 (N–H), 3004 (aromatic = C–H), 2943 (aliphatic C–H), 1704 (C=O), 1602, 1595 (aromatic C=C), 1462, 1443 (aliphatic C-H), 1360, 1251 (aliphatic C–C), 1121, 1058 (C–N) cm^-1^. ^1^H NMR (d_6_-DMSO, 500 MHz): δ = 1.87–1.89 (m, 2H, aliphatic CH_2_), 2.12–2.15 (m, 2H, aliphatic CH_2_), 2.68–2.77 (m, 2H, aliphatic CH_2_), 3.79 (s, 3H, N–CH_3_), 6.55 (d, J = 8.6 Hz, 1H, Ar H), 6.59 (d, J = 7.2 Hz, 2H, Ar H), 7.07 (t, J = 7.4 Hz, 2H, Ar H), 7.20 (t, J = 7.1 Hz, 1H, Ar H), 7.66 (s, 1H, Ar H), 7.97 (d, J = 7.0 Hz, 1H, Ar H), 10.27 (s, 1H, NH), 10.48 (s, 1H, NH). ^13^C NMR (d_6_-DMSO, 126 MHz): δ = 21.43 (aliphatic CH_2_), 28.14 (aliphatic CH_2_), 35.73 (aliphatic CH_2_), 37.26 (N-CH_3_), 49.53 (quarternary C), 99.35 (Ar C), 108.29 (Ar C), 108.86 (Ar C), 118.72 (Ar C), 125.16 (Ar C), 127.78 (Ar C), 128.09 (Ar C), 128.62 (Ar C), 128.89 (Ar C), 133.44 (Ar C), 137.71 (Ar C), 139.54 (Ar C), 142.14 (Ar C), 147.50 (Ar C), 155.50 (Ar C), 180.77 (C = O), 193.97 (C = O). MS: *m/z* (ESI): 441, 442; Anal. Calcd for C_24_H_19_N_5_O_4_: C, 65.30; H, 4.34; N, 15.86; O, 14.50; Found: C, 65.47; H, 4.23; N, 15.71%.

### 1′,7′,7′-trimethyl-5-nitro-3′-phenyl-6′,7′,8′,9′-tetrahydrospiro[indoline-3,4′-pyrazolo[3,4-*b*]quinoline]-2,5′(1′*H*)-dione (6f)

White powder, 0.398 g (85%). Mp: 294–297 °C. FTIR (ATR): v = 3646 (N–H), 3318 (N–H), 3017 (aromatic = C–H), 2955 (aliphatic C–H), 1695 (C=O), 1605, 1597 (aromatic C=C), 1475, 1447 (aliphatic C–H), 1337, 1283 (aliphatic C–C), 1190, 1094 (C–N) cm^−1^. ^1^H NMR (d_6_-DMSO, 500 MHz): δ = 1.00 (s, 3H, aliphatic CH_3_), 1.02 (s, 3H, aliphatic CH_3_), 2.02–2.03 (m, 2H, aliphatic CH_2_), 2.55–2.65 (m, 2H, aliphatic CH_2_), 3,79 (s, 3H, N-CH_3_), 6.56 (d, *J* = 8.6 Hz, 1H, Ar H), 6.60 (d, *J* = 7.8 Hz, 2H, Ar H), 7.07 (t, *J* = 7.5 Hz, 2H, Ar H), 7.20 (t, *J* = 7.2 Hz, 1H, Ar H), 7.63 (dd, *J*_*1*_ = 7.2 Hz, *J*_*2*_ = 2.0 Hz, 1H, Ar H), 7.96–7.98 (dd, *J*_*1*_ = 8.5 Hz, *J*_*2*_ = 2.0 Hz, 1H, Ar H), 10.23 (s, 1H, NH), 10.50 (s, 1H, NH). ^13^C NMR (d_6_-DMSO, 126 MHz): δ = 27.79 (aliphatic CH_3_), 28.02 (aliphatic CH_3_), 32.66 (aliphatic C(CH_3_)_2_), 35.73 (N-CH_3_), 49.42 (aliphatic CH_2_), 50.69 (aliphatic CH_2_), 56.46 (quarternary C), 99.29 (Ar C), 107.00 (Ar C), 108.92 (Ar C), 118.49 (Ar C), 125.20 (Ar C), 127.80 (Ar C), 128.10 (Ar C), 128.87 (Ar C), 130.62 (Ar C), 133.44 (Ar C), 137.84 (Ar C), 139.46 (Ar C), 142.10 (Ar C), 147.50 (Ar C), 149.36 (Ar C), 153.55 (Ar C), 180.69 (C=O), 193.68 (C=O). MS: *m/z* (ESI): 469, 470; Anal. Calcd for C_26_H_23_N_5_O_4_: C, 66.51; H, 4.94; N, 14.92; O, 13.63; Found: C, 66.64; H, 5.03; N, 14.98%.

### 1-methyl-5′-nitro-3-phenyl-6,7-dihydro-1*H*-spiro[cyclopenta[*e*]pyrazolo[3,4-*b*]pyridine-4,3′-indoline]-2′,5(8*H*)-dione (6g)

White powder, 0.388 g (91%). Mp: 277–280 °C. FTIR (ATR): v = 3479 (N–H), 3310 (N–H), 3075, 3025 (aromatic = C–H), 2968, 2934 (aliphatic C–H), 1701, 1667 (C=O), 1612, 1606 (aromatic C=C), 1479, 1448 (aliphatic C–H), 1329, 1297 (aliphatic C=C), 1179, 1067 (C–N) cm^-1^. ^1^H NMR (d_6_-DMSO, 500 MHz): δ = 2.25–2.27 (m, 2H, aliphatic CH_2_), 2.74–2.84 (m, 2H, aliphatic CH_2_), 3.82 (s, 3H, N–CH_3_), 6.71–6.78 (m, 3H, Ar H), 7.02–7.10 (m, 2H, Ar H), 7.17 (t, *J* = 7.3 Hz, 1H, Ar H), 7.68 (d, *J* = 1.8 Hz, 1H, Ar H), 8.02 (dd, J_1_ = 8.6 Hz, *J*_*2*_ = 2.0 Hz, 1H, Ar H), 10.88 (s, 1H, NH), 11.03 (s, 1H, NH) ppm. ^13^C NMR (d_6_-DMSO, 126 MHz): δ = 24.66 (aliphatic CH_2_), 33.81 (aliphatic CH_2_), 35.77 (N-CH_3_), 48.08 (quarternary C), 99.06 (Ar C), 109.40 (Ar C), 112.29 (Ar C), 119.78 (Ar C), 123.42 (Ar C), 125.75 (Ar C), 128.06 (Ar C), 128.21 (Ar C), 128.74 (Ar C), 133.43 (Ar C), 137.37 (Ar C), 140.35 (Ar C), 142.50 (Ar C), 143.81 (Ar C), 147.66 (Ar C), 148.99 (Ar C), 167.41 (Ar C), 179.53 (C = O), 199.49 (C = O) ppm. MS: *m/z* (ESI): 427, 428; Anal. Calcd for C_23_H_17_N_5_O_4_: C, 64.63; H, 4.01; N, 16.39; O, 14.97; Found: C, 64.52; H, 4.18; N, 16.45%.

### 1-methyl-5′-nitro-3-phenyl-1*H*-spiro[indeno[2,1-*e*]pyrazolo[3,4-*b*]pyridine-4,3′-indoline]-2′,5(10*H*)-dione (6h)

Red powder, 0.441 g (93%). Mp: > 300 °C. FTIR (ATR): v = 3207, 3161 (N–H), 3032, 3013 (aromatic = C–H), 2942 (aliphatic C–H), 1737, 1695 (C=O), 1659, 1622 (aromatic C=C), 1494, 1448 (aliphatic C–H), 1338, 1231 (aliphatic C–C), 1159, 1073 (C-N) cm^-1^. ^1^H NMR (d_6_-DMSO, 500 MHz): δ = 3.97 (s, 3H, N-CH_3_), 6.76–6.80 (m, 2H, Ar H), 7.09 (m, 2H, Ar H), 7.19 (m, 2H, Ar H), 7.37 (t, J = 7.3 Hz, 1H, Ar H),7.52 (t, J = 7.3 Hz, 1H, Ar H), 7.81–7.85 (m, 2H, Ar H), 7.93–8.05 (m, 2H, Ar H), 11.00 (s, 1H, NH), 11.36 (s, 1H, NH) ppm. ^13^C NMR (d_6_-DMSO, 126 MHz): δ = 36.45 (N–CH_3_), 47.68 (quarternary C), 100.57 (Ar C), 104.81 (Ar C), 109.64 (Ar C), 120.13 (Ar C), 120.65 (Ar C), 120.93 (Ar C), 123.33 (Ar C), 125.94 (Ar C), 128.13 (Ar C), 129.75 (Ar C), 131.14 (Ar C), 132.38 (Ar C), 133.20 (Ar C), 136.40 (Ar C), 136.89 (Ar C), 137.28 (Ar C), 139.88 (Ar C), 142.07 (Ar C), 142.71 (Ar C), 147.92 (Ar C), 148.81 (Ar C), 149.95 (Ar C), 156.88 (Ar C), 179.66 (C=O), 189.18 (C=O) ppm. MS: *m/z* (ESI): 475, 476; Anal. Calcd for C_27_H_17_N_5_O_4_: C, 68.21; H, 3.60; N, 14.73; O, 13.46; Found: C, 68.28; H, 3.66; N, 14.62%.

### Microdilution method

The antimicrobial study was carried out using the microdilution (MIC) method^74,75^. In this study, besides using *Staphylococcus aureus* ATCC 25,923 (*S.aureus*) and *Enterococcus faecalis* (*E.faecalis*) as bacteria, *Candida albicans* ATCC 10,231 (*C.albicans*) was used as yeast. The stock solutions of the complexes were prepared in DMSO and used in all stages of the study. Microorganisms were grown in 5 ml of Nutrient Broth (NB) at 37 °C for 18 h in a shaker incubator. It was taken from the grown bacteria and yeast cells and added to 50 ml of NB. Afterword 10^6^ bacteria per ml were obtained in accordance with the 0.5 McFarland turbidity standard. Followed by serial dilution and the serial dilution tubes are incubated at 37 °C for 24 h. The last tube without bacterial growth is accepted as the MIC values. The MIC values were given in μg/ml. Gentamicin and flucanazole were used as control group.

### Time-kill kinetics assay

After the bacteria were grown in broth (NB) at 37 °C for overnight, they were added to the other sterile broth medium at a rate of approximately 10^5^ microorganisms per ml. MIC, MICx2, MICx4 were added to the media prepared separately from the compounds with previously determined MIC values. Colony counts were made on Nutrient Broth medium by taking samples at regular intervals for 24 h from the samples placed in the incubator at 37 °C.

The colony counts were converted into logarithmic values and presented in graphs and Table 5. In addition, the *t*_*99*_ values as obtained from the colony counts were defined and the life span of the bacteria was determined. The value *t*_*99*_ is calculated as the time corresponding to 2 logarithmic reductions during the 24-h period^76^. Compounds 4b, 4h and 6h which were the most effective ones compared to the MIC values were used in the study.

### Free radical scavenging activity

The free radical scavenging activity was determined by the 1,1-diphenyl-2-picryl-hydrazyl (DPPH•). The activity was measured by following the methodology described by Brand-Williams et al.^77^. Briefly, 20 mg/L DPPH• in methanol was prepared and 1.5 mL of this solution was added to 0.75 mL of synthesized compounds solution in methanol at different concentrations (10–400 μg/mL). After 30 min the absorbance was measured at 517 nm. Methanol (0.75 mL) in place of the sample was used as control. Lower absorbance of the reaction mixture indicates higher free radical scavenging activity. The percent inhibition activity was calculated using the following equation:

$${\text{Free radical scavenging effect }}\% = [(A_{0} - A_{1} )/A_{0} ]\times 100.$$

(*A*_*0*_ = the control absorbance and *A*_*1*_ = the sample solution absorbance).

### Molecular docking

3V7R and 3E5P were selected as potential target proteins for the antimicrobial activity, and 6LU7 protein for the Sar-Cov-2 main protease inhibition. Known information on the PDB: 3V7R protein shows that the binding strategy of the biotin ligase enzyme of the Staphylococcus aureus protein could be selectively inhibited^78^. The 3E5P as a member of the Alanine racemase (AlaR) is a ubiquitous bacterial enzyme and provides the conversion between L- and D-alanine as the essential cell wall precursor^79^. N3 can specifically, selectivity inhibits Mpro from SARS-CoV and MERS-CoV and has exhibited potent antiviral activity against infectious bronchitis virus^80^.

The crystalline structures of the 32G ((3aS,4S,6aR)-4-(5-{1-[4-(6-amino-9H-purin-9-yl)butyl]-1H-1,2,3-triazol-4-yl}pentyl) complex were downloaded from the PDB IDs 3V7R protein database^81^. 3D crystal structure of the PRD_002214 (N-[(5-methylisoxazol-3-yl)carbonyl]alanyl-l-valyl-n ~ 1 ~ -((1r,2z)-4-(benzyloxy)-4-oxo-1-{[(3r)-2-oxopyrrolidin-3-yl]methyl}but-2-enyl)-l-leucinamide) with SARS-CoV-2 (PDB ID: 6LU7) complex were downloaded from research collaboratory for structural bioinformatics protein data bank (RCSB PDB).

Firstly, each related proteins have been identified as the receptors, the complexed ligands, water as a non-amino acid residues were manually removed from the target proteins using the Discovery Studio Client (Dassault Systems BIOVIA2021), and added hydrogen atoms^82^.

The molecular structure of each compound was drawn accurately using ChemDraw Ultra 12.0, then optimized with the Gaussian G09 program based on density functional level of theory at Becke’s three-parameter Lee–Yang–Parr hybrid functional as B3LYP/6.311 G(d.p) basic set in a vacuum^83,84^. Through this process was reached the lowest energy or optimized structure of the molecule.

As part of this work, all docking studies were performed using the Autodock 4^85^ with the Lamarckian Genetic Algorithm and ten different runs configured to finish after a maximum of 250,000 number of energy assessments were used to obtain the results of the docking experiment.

**Figure 9:**
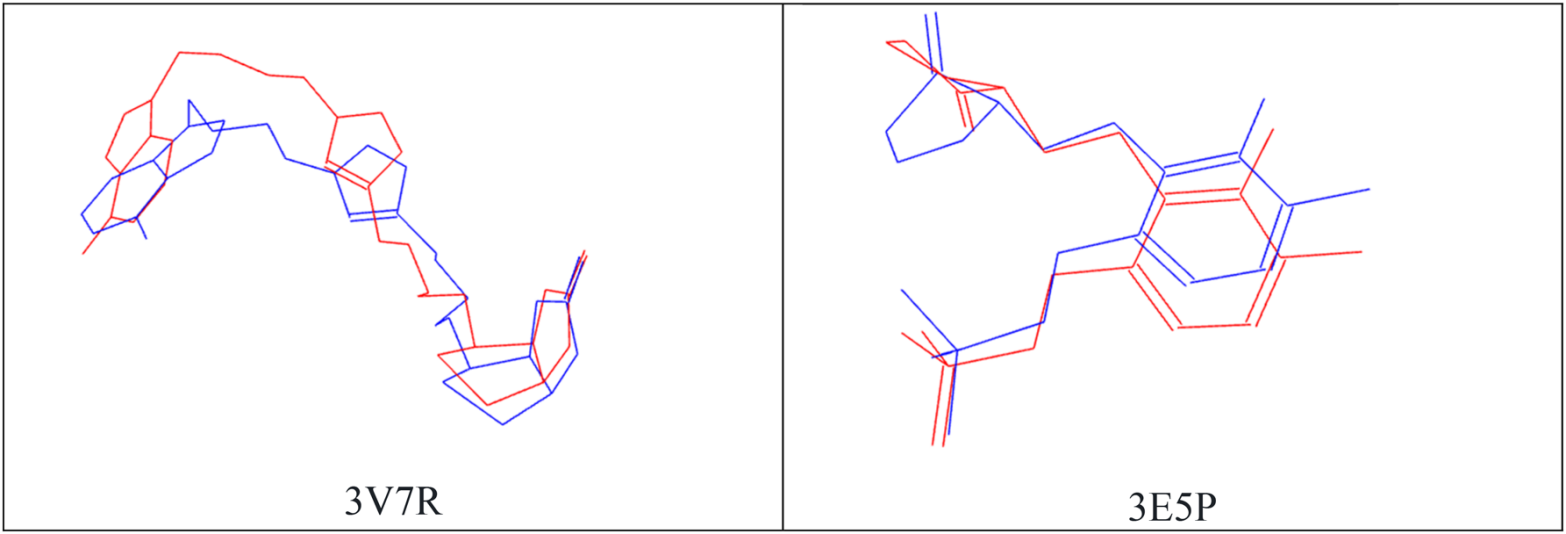
Validation of the 3V7R and 3E5P proteins, red is native ligand conformation before docking process and blue is native ligand conformation after docking process.

For the docking validation, simulation of ligand–protein interactions was carried out within Lamarckian genetic algorithms methods and the grid box was set at 40 × 40 × 40 Å in the x, y, z directions with a spacing of 0.375 Å in the active site centre. Each co-crystallized ligand was previously removed from each protein binding site. We compared the predicted docking pose with the experimental co-crystallized binding pose. The small RMSD variation (1.84 Å) was obtained from the re-docking calculations of 6LU7 native ligand, suggesting that the program could correctly and efficiently simulate the experimental results for the respective ligands. The 3V7R receptor crystal with the specified ligands as 32G 3D-crystal was simulated re-docking and the RMSD value was obtained 0.72 Å under this condition. In order to validate the docking approach for the 3E5P protein structure used, the respective co-crystallized ligand, named DCS, was docked to the protein’s active site and docking validation results of the root mean square deviation was obtained 0.82 Å (Fig. 9).

## Supplementary Information

Supplementary Figures.

## Data Availability

The data used to support the findings of this study are included within the article and supplementary materials.
