# Supplementary material for: Synthesis, antimicrobial activity and molecular docking studies of spiroquinoline-indoline-dione and spiropyrazolo-indoline-dione derivatives
Source: Sci Rep. 2023 Jan 30;13:1676. doi: 10.1038/s41598-023-27777-z (PMC9885930; doi:10.1038/s41598-023-27777-z)

SUPPLEMENTARY MATERIAL

SYNTHESIS OF ISATIN-BASED SPIROQUINOLINE-INDOLINE-DIONE AND SPIROPYRAZOLO-INDOLINE-DIONE DERIVATIVES: ANTIMICROBIAL, ANTIOXIDANT ACTIVITY AND THEIR MOLECULAR DOCKING STUDIES

Melek Gul[1], Emine Turk Celikoglu[2], Onder Idil[3], Gamze Tas[4], Emel Pelit[4*]

[1]Department of Chemistry, Faculty of Art and Sciences, Amasya University, 05100, Amasya, Turkey

[2]Department of Biology, Faculty of Art and Sciences, Amasya University, 05100, Amasya, Turkey

[3]Department of Pre-School Education, Faculty of Education, Amasya University, 05100, Amasya, Turkey

[4]Department of Chemistry, Faculty of Art and Sciences, Kirklareli University, 39100, Kirklareli, Turkey

^{1}H and ^{13}C NMR spectrums of compounds 4a-h and 6a-h

^{1}H and ^{13}C NMR spectrums of Compound 4a

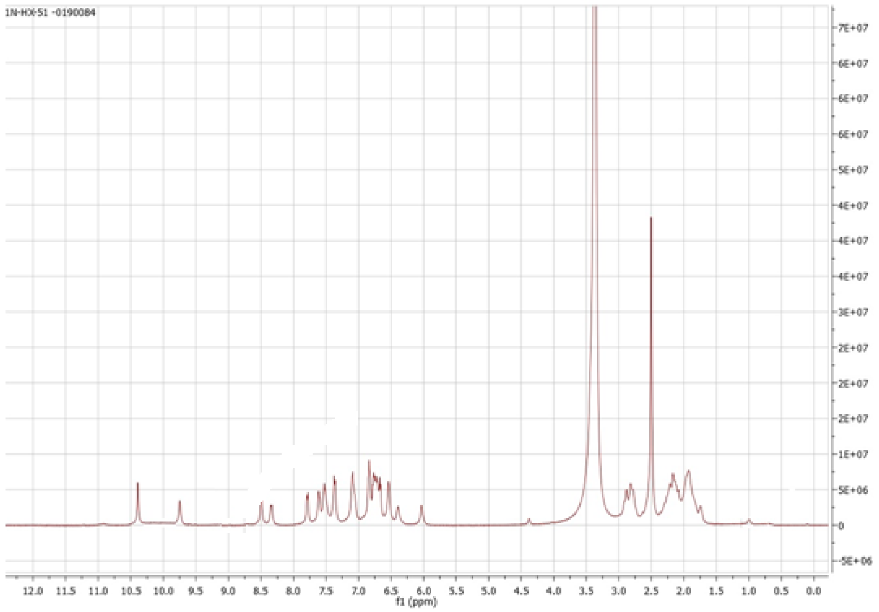

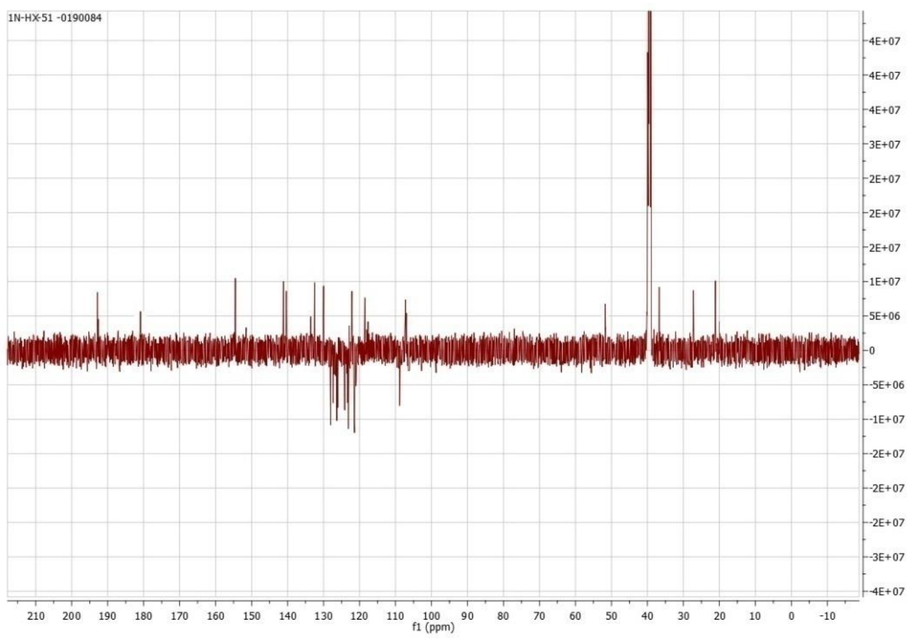

^{1}H and ^{13}C NMR spectrums of Compound 4b

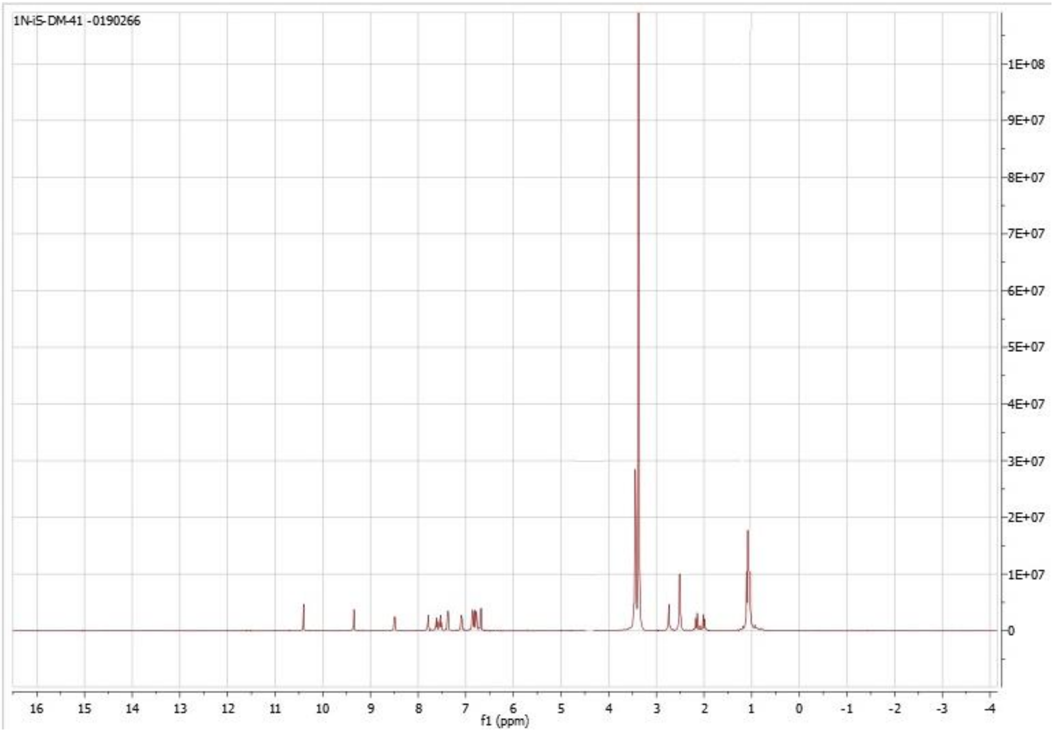

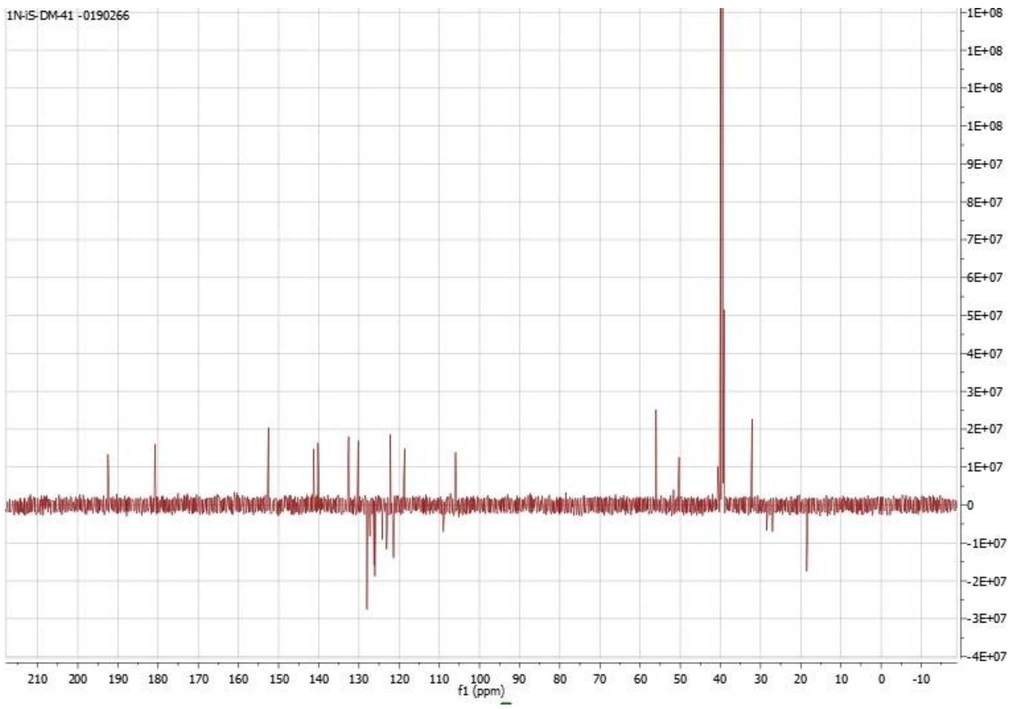

^1H and ^{13}C NMR spectrums of Compound 4c

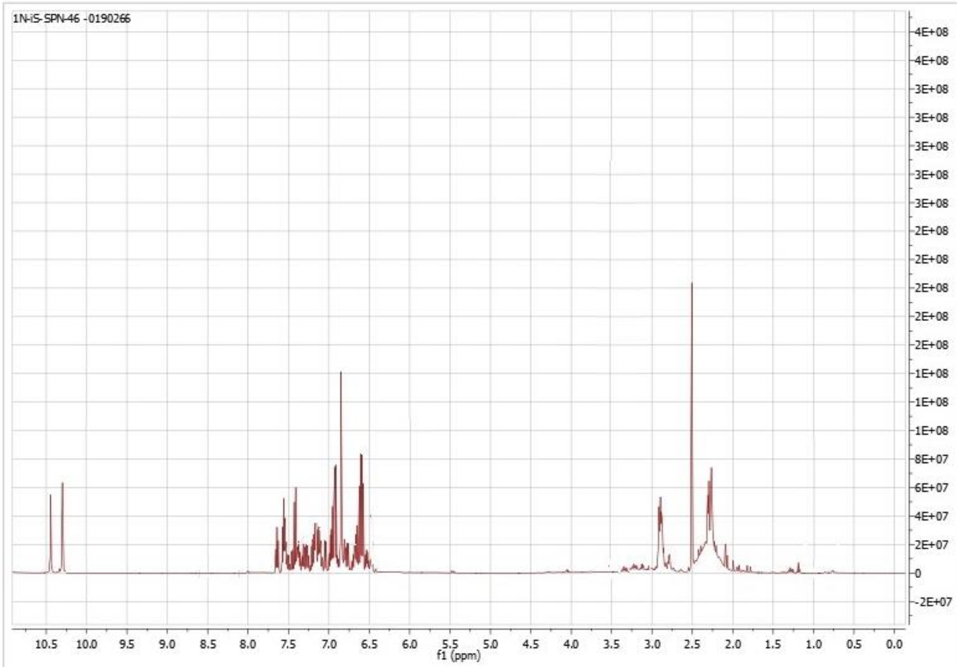

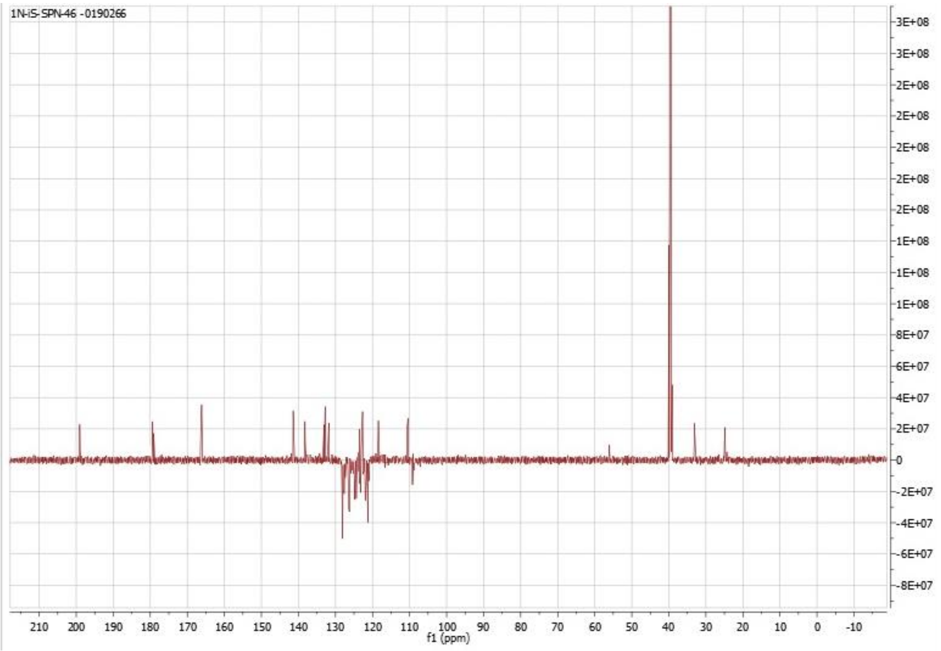

^{1}H and ^{13}C NMR spectrums of Compound 4d

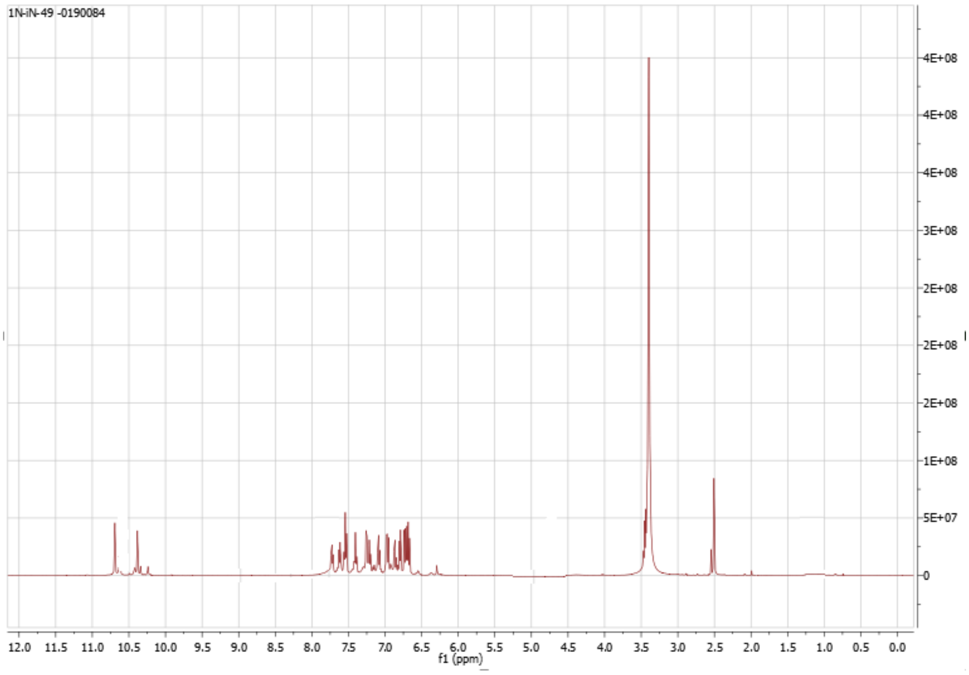

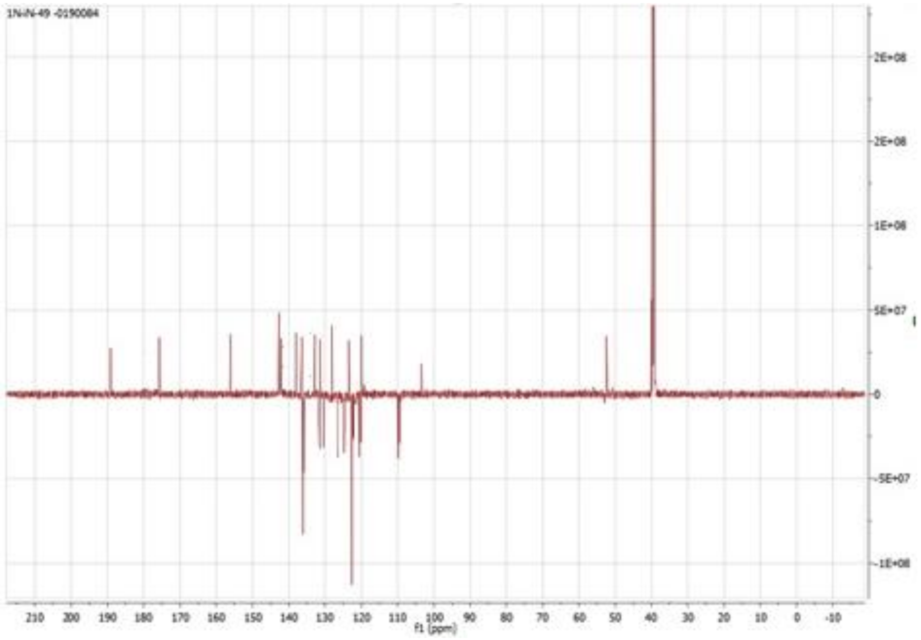

^{1}H and ^{13}C NMR spectrums of Compound 4e

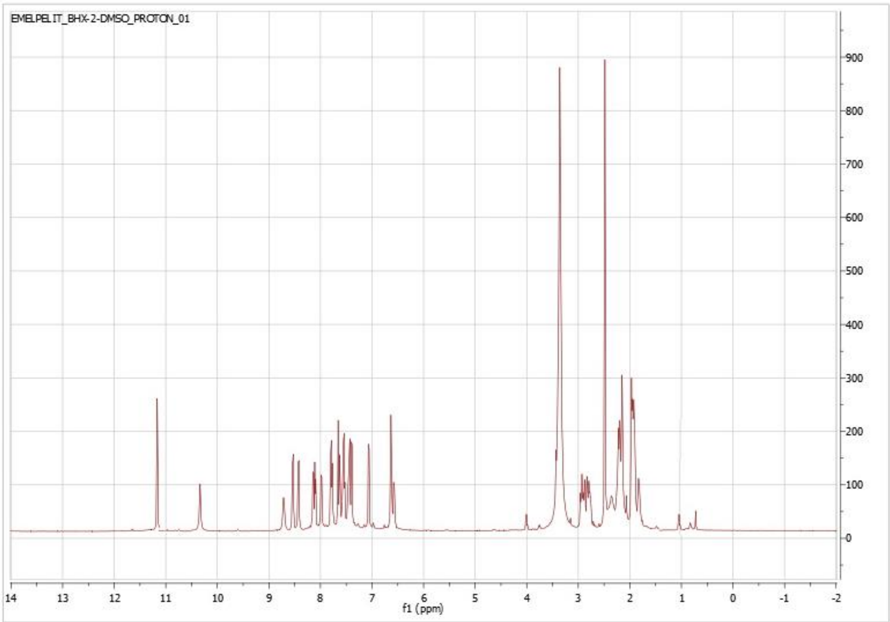

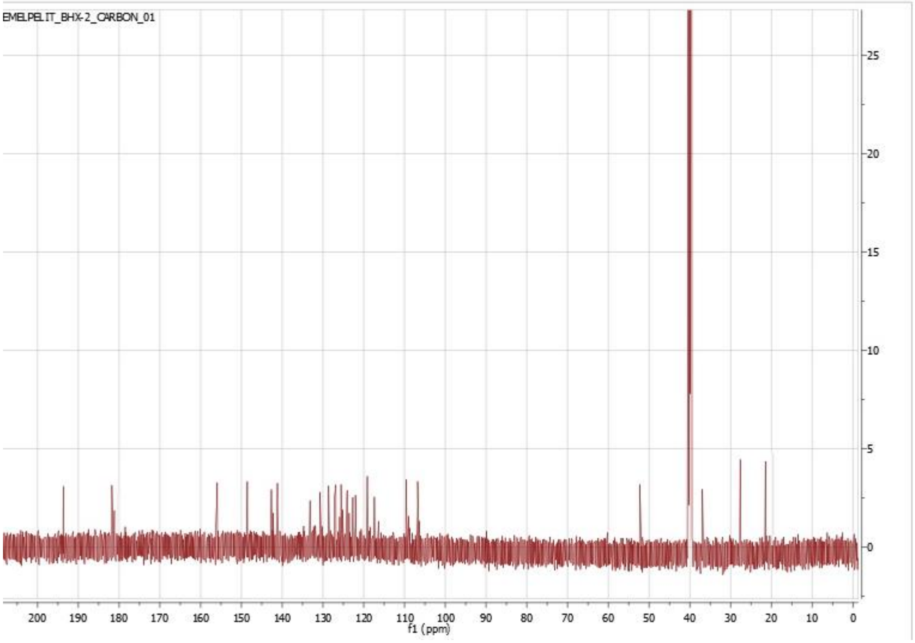

^{1}H and ^{13}C NMR spectrums of Compound 4f

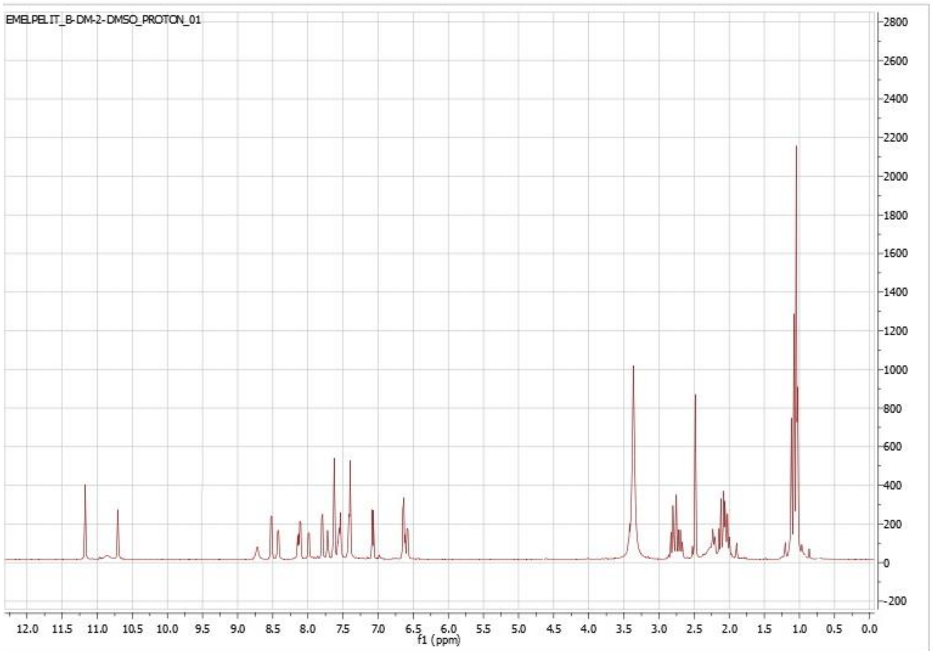

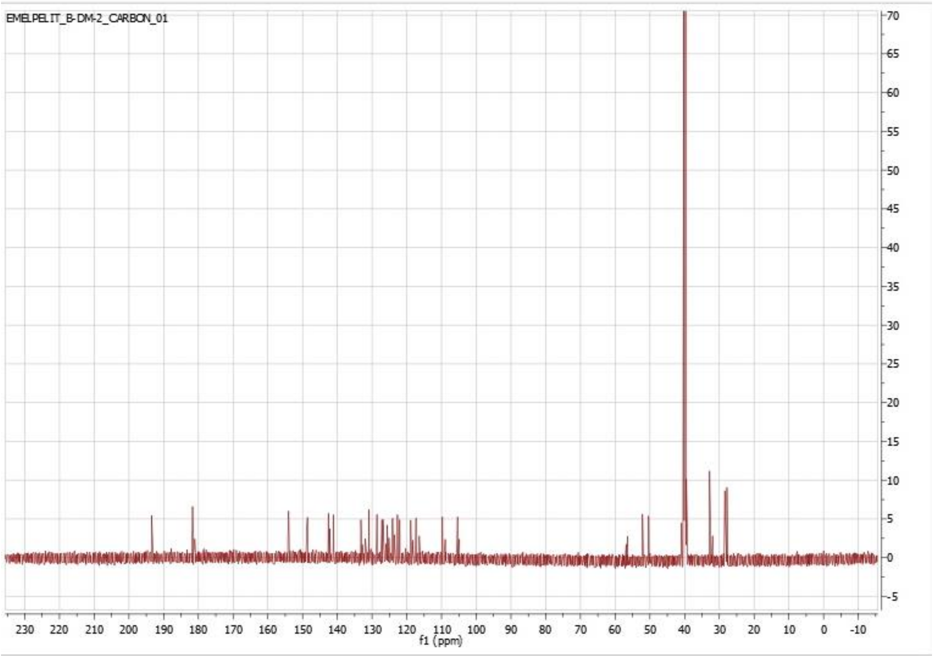

^{1}H and ^{13}C NMR spectrums of Compound 4g

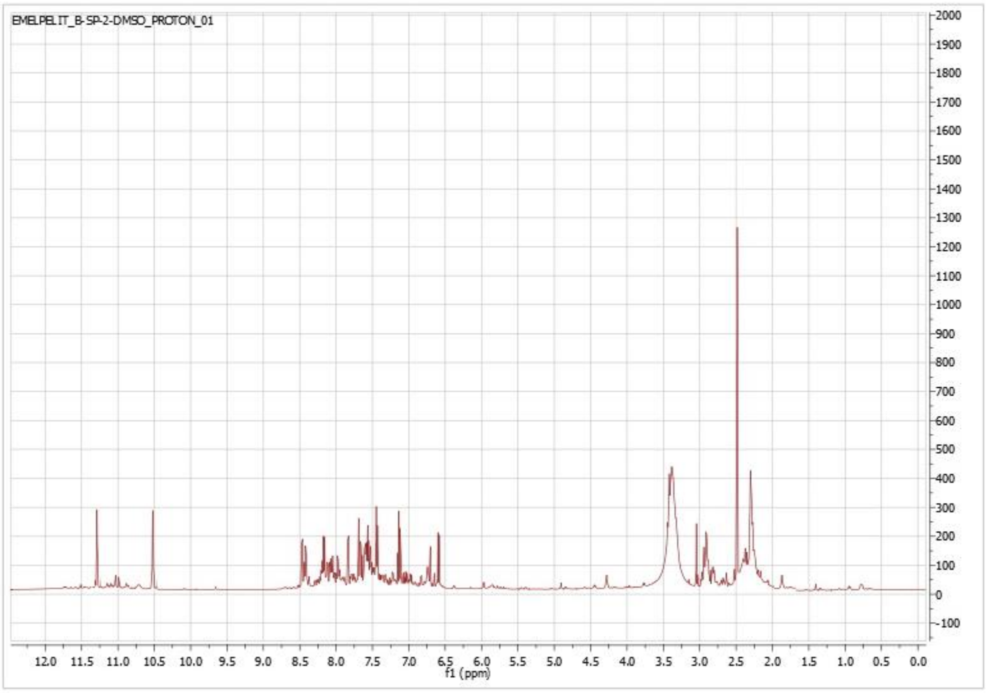

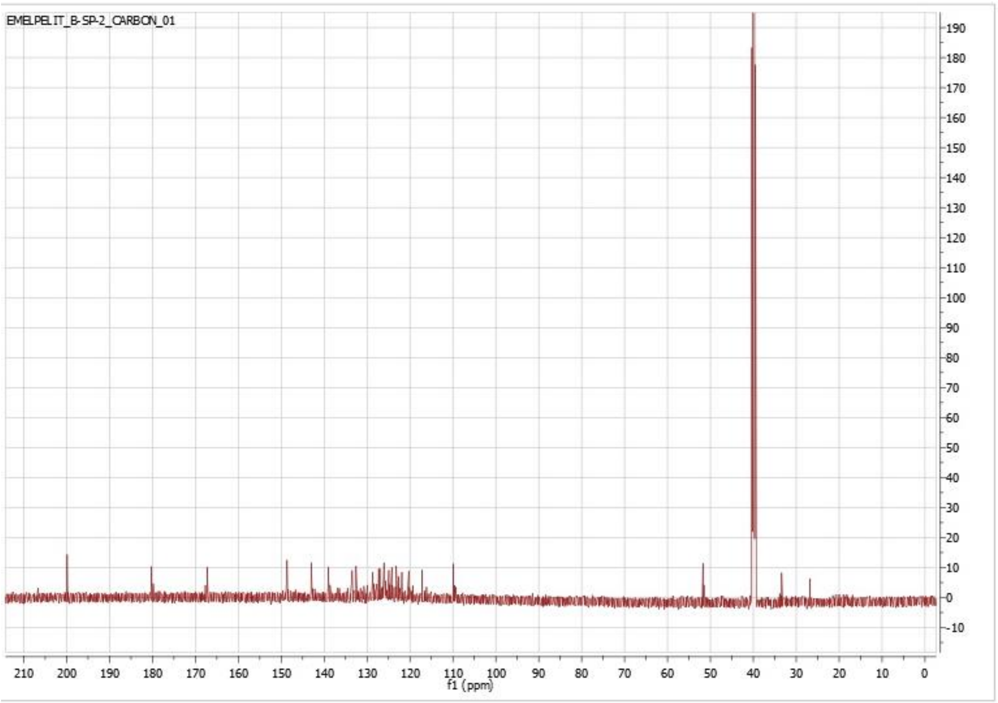

^{1}H and ^{13}C NMR spectrums of Compound 4h

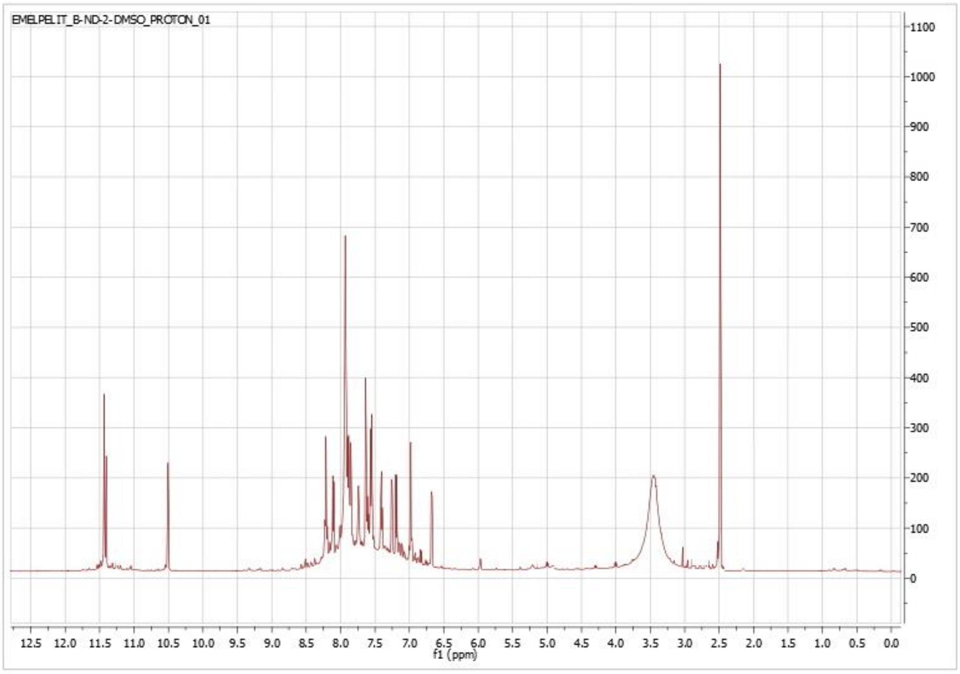

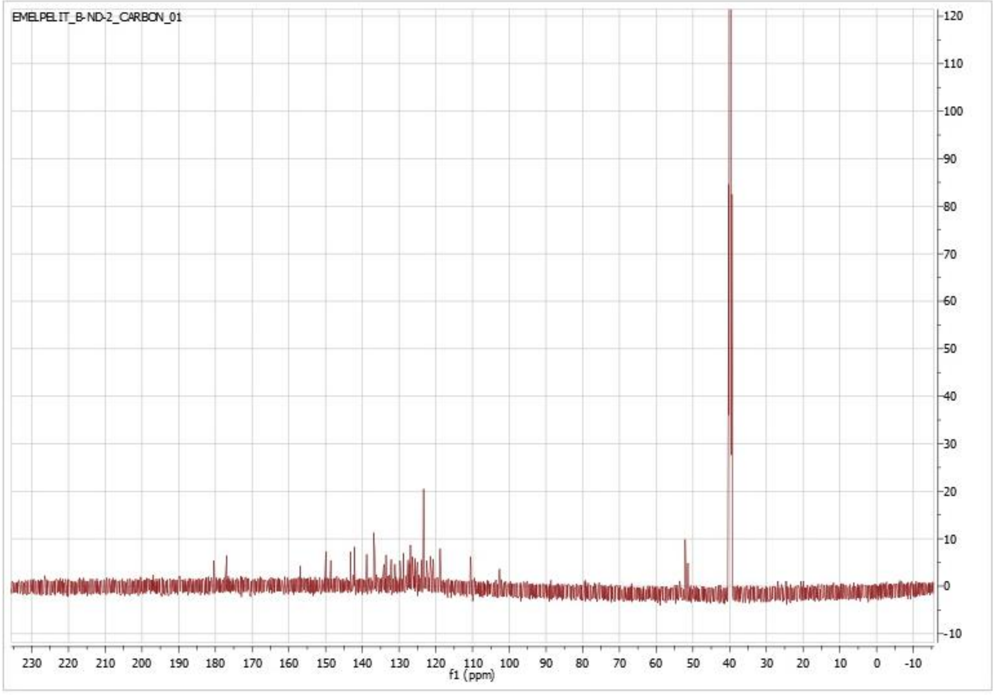

^{1}H and ^{13}C NMR spectrums of Compound 6a

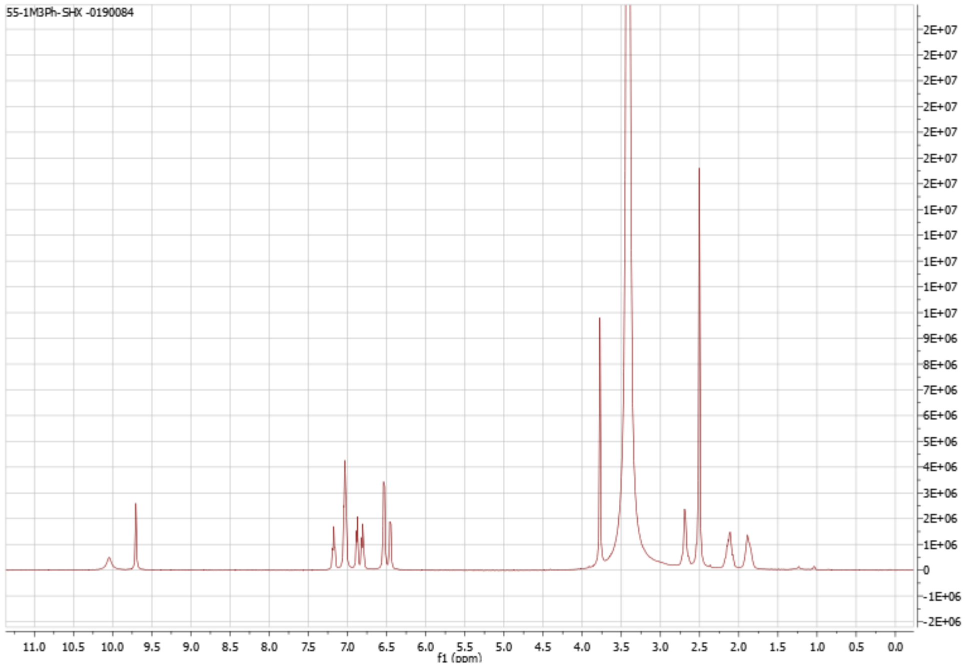

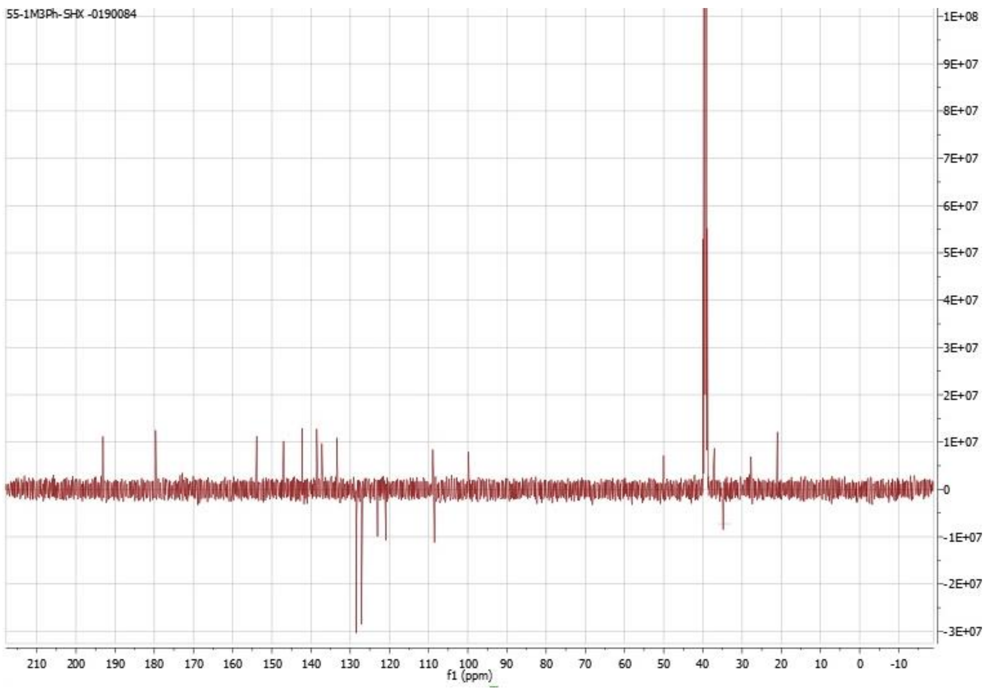

^{1}H and ^{13}C NMR spectrums of Compound 6b

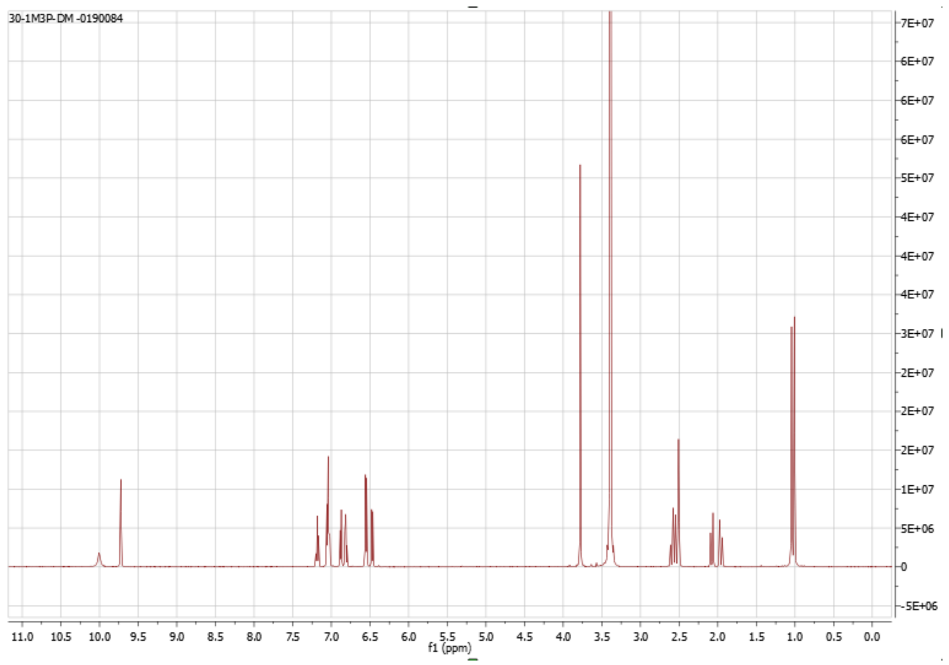

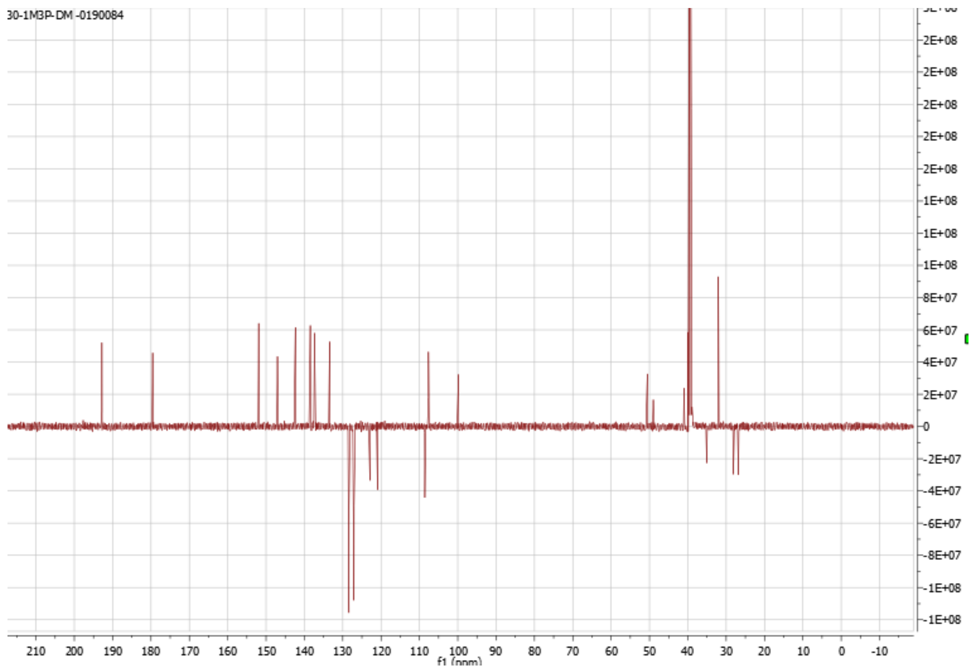

^{1}H and ^{13}C NMR spectrums of Compound 6c

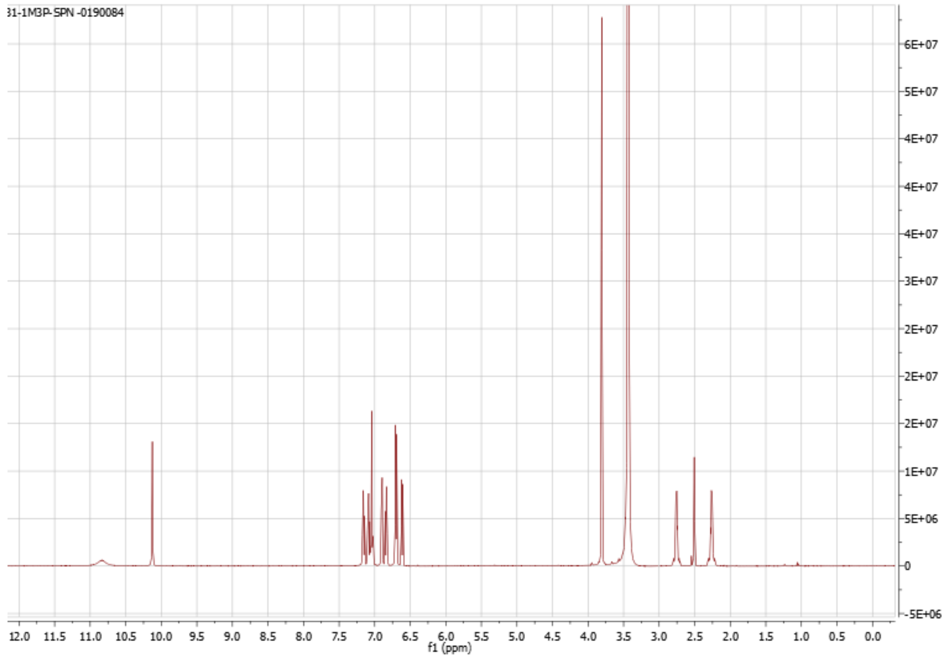

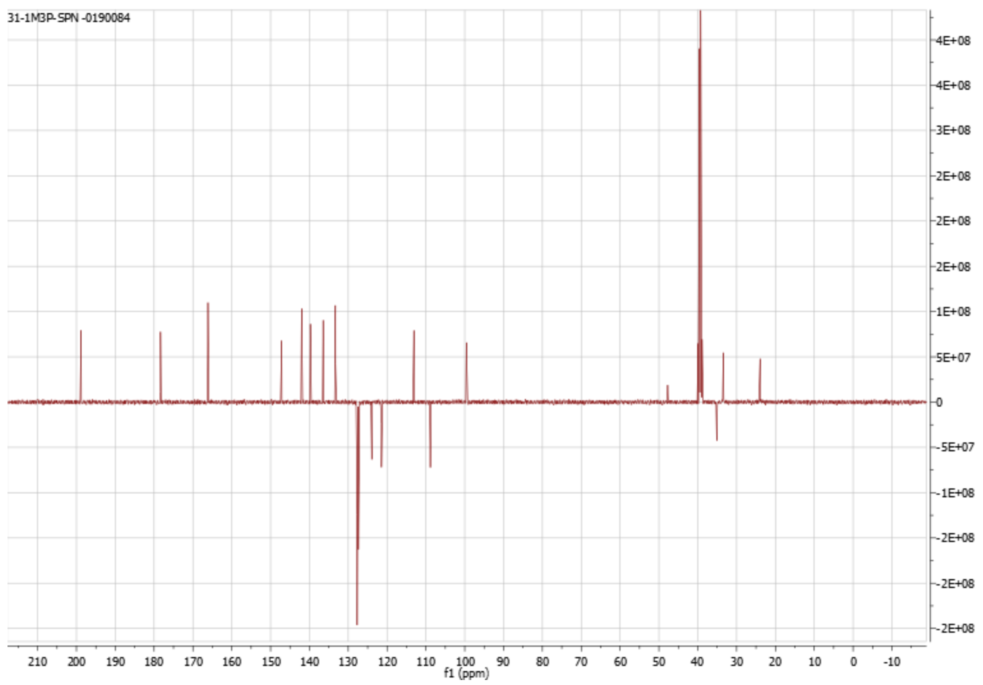

^{1}H and ^{13}C NMR spectrums of Compound 6d

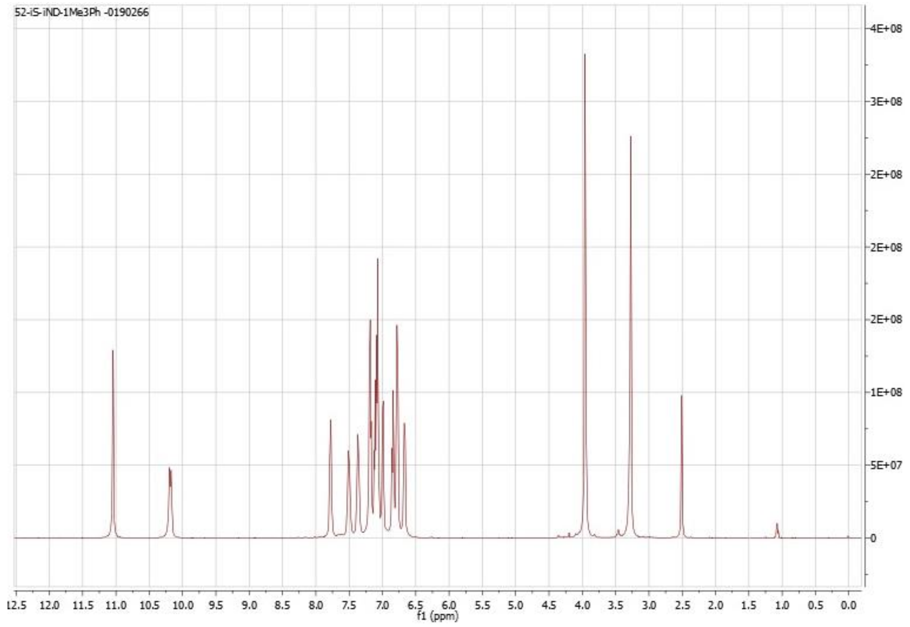

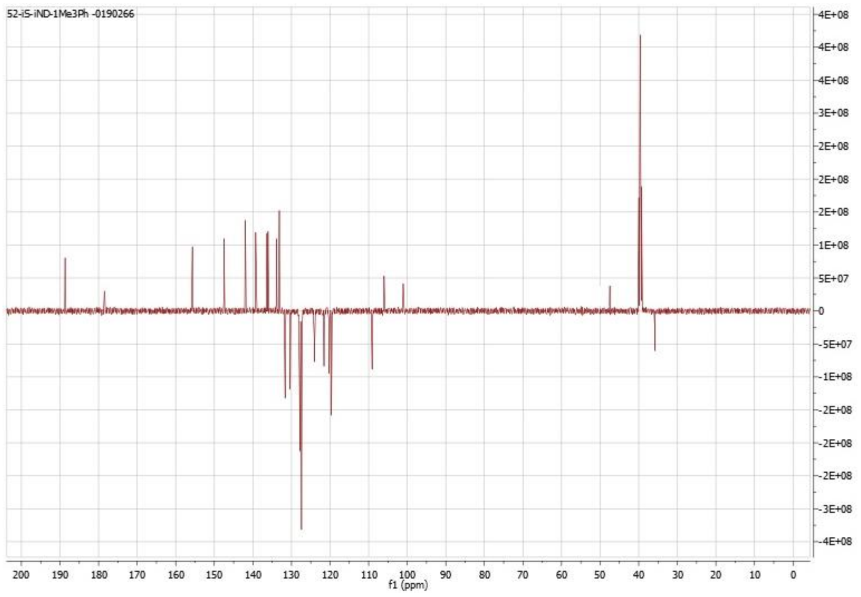

^{1}H and ^{13}C NMR spectrums of Compound 6e

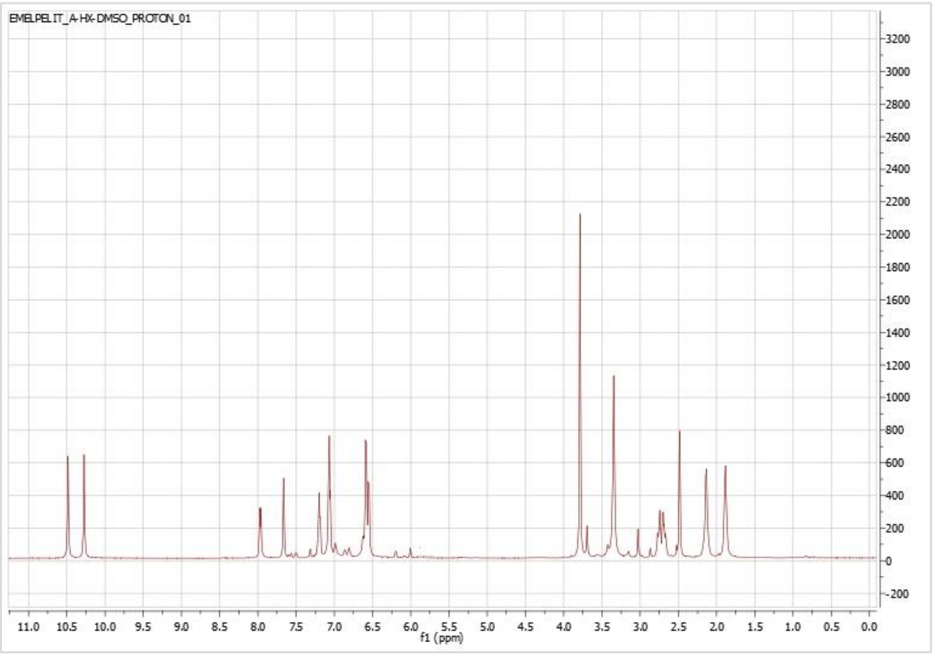

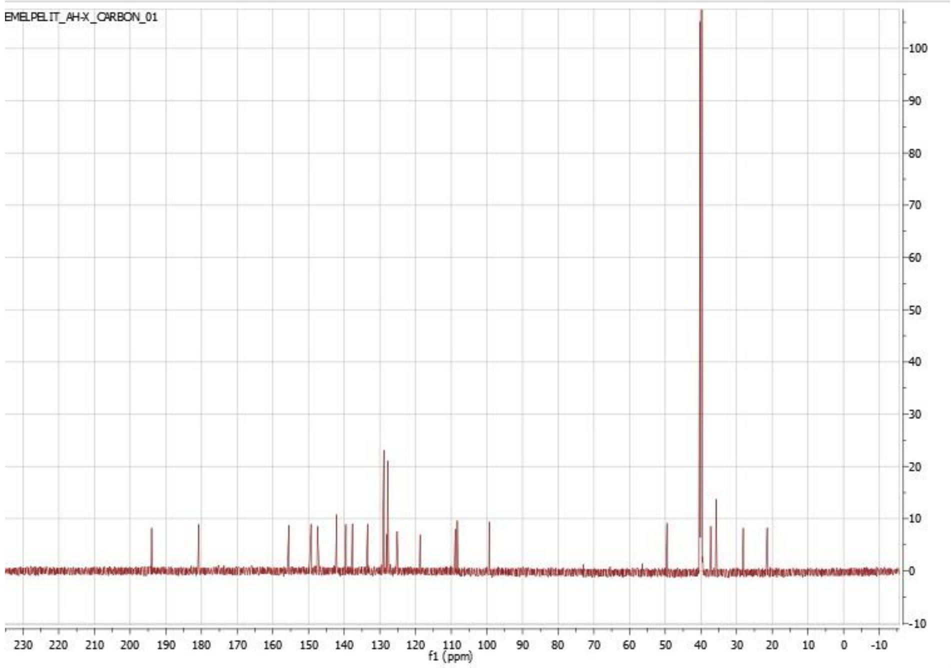

^{1}H and ^{13}C NMR spectrums of Compound 6f

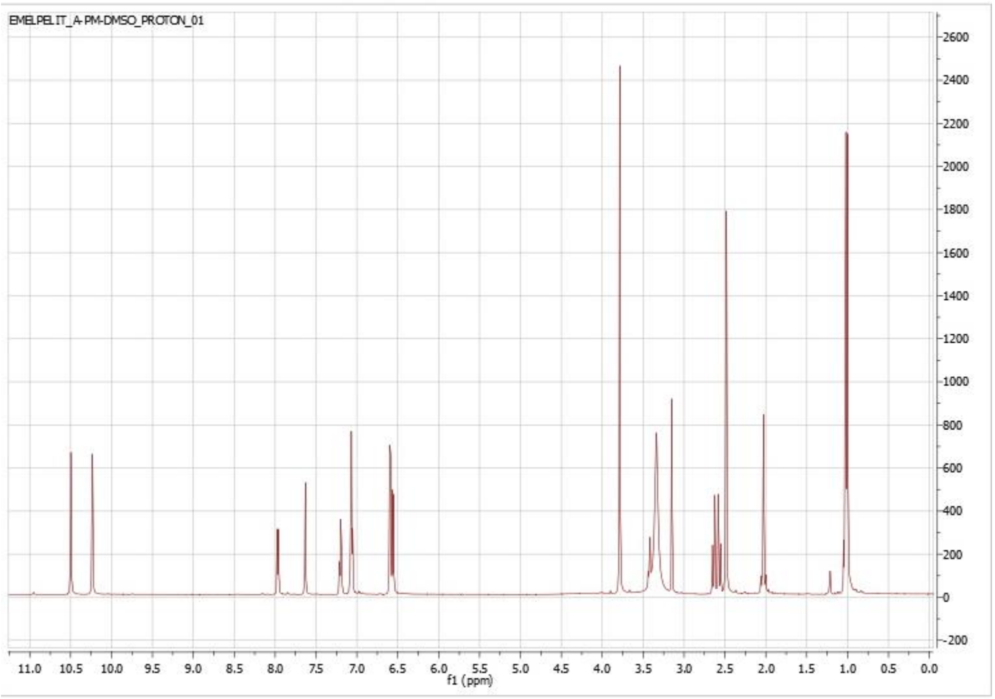

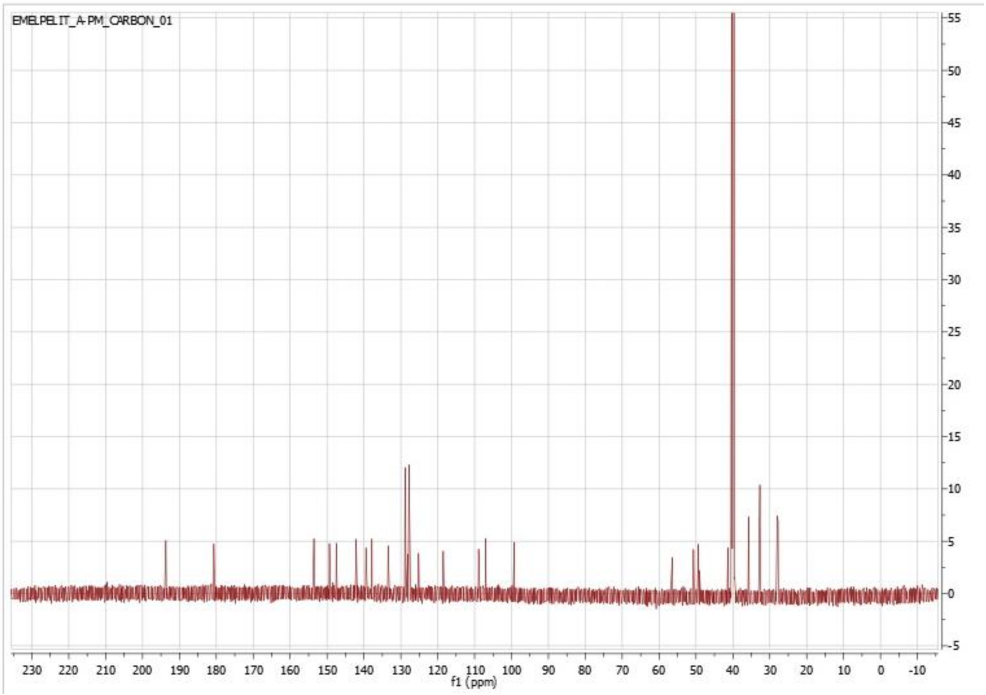

^{1}H and ^{13}C NMR spectrums of Compound 6g

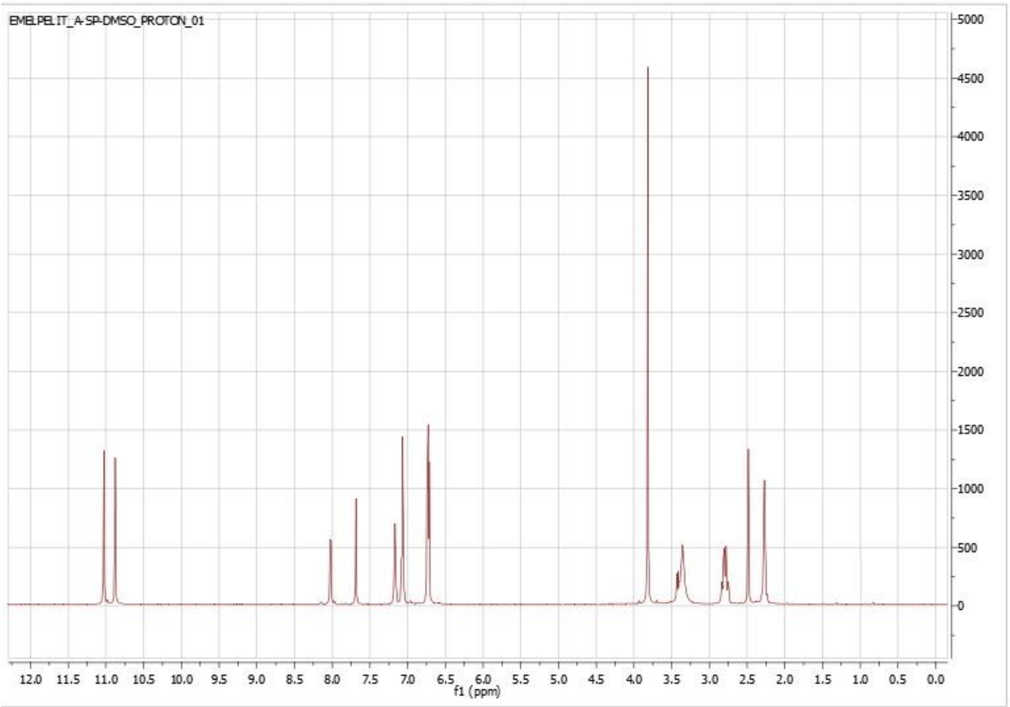

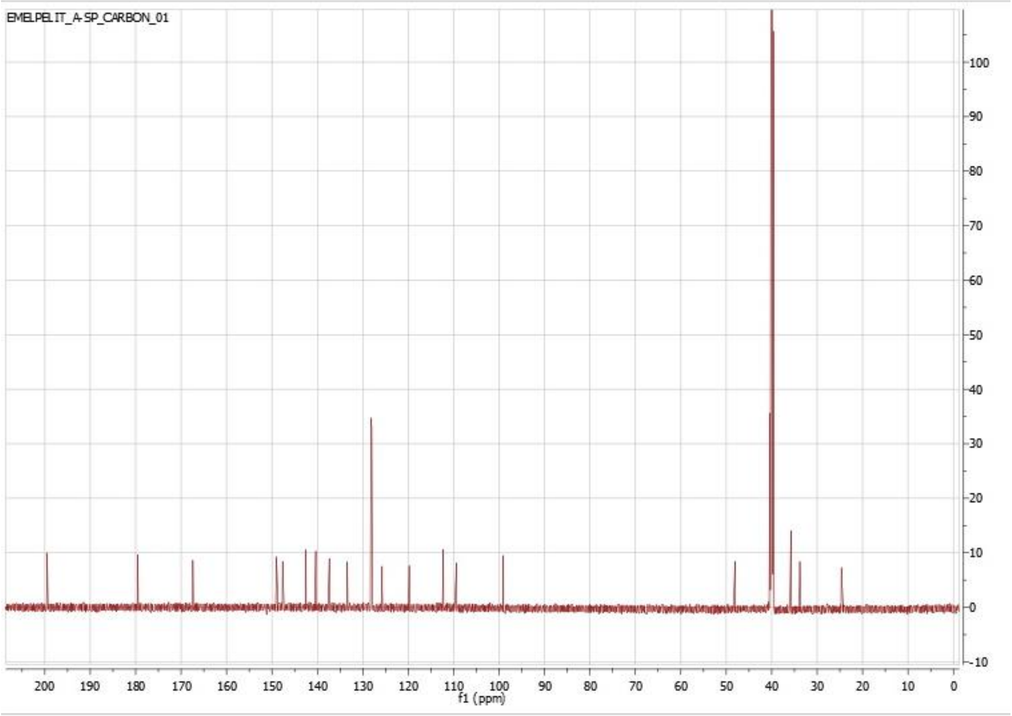

^1H and ^{13}C NMR spectrums of Compound 6h

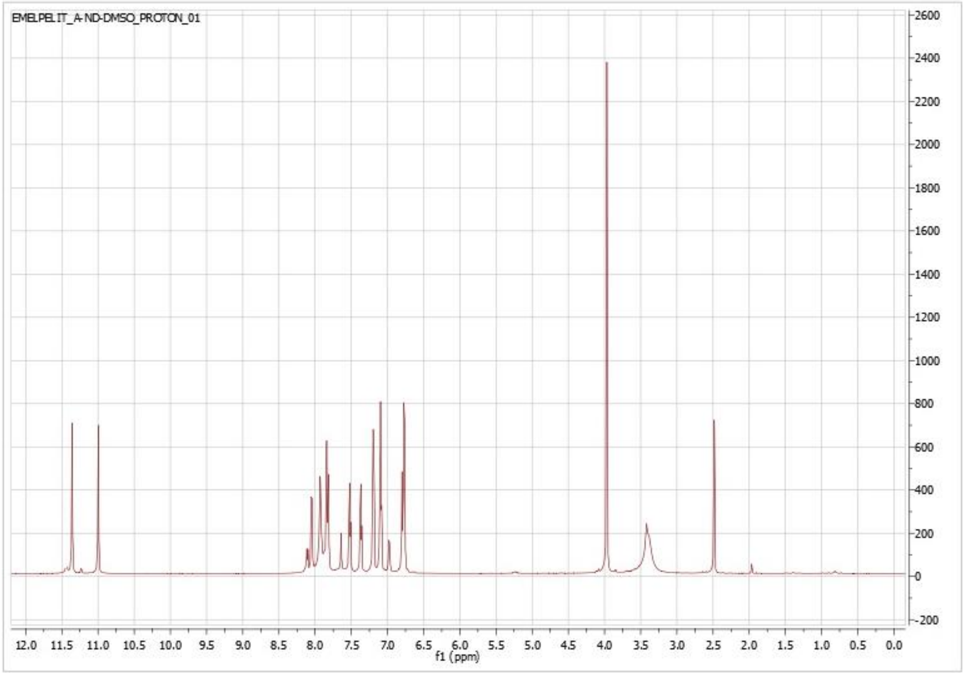

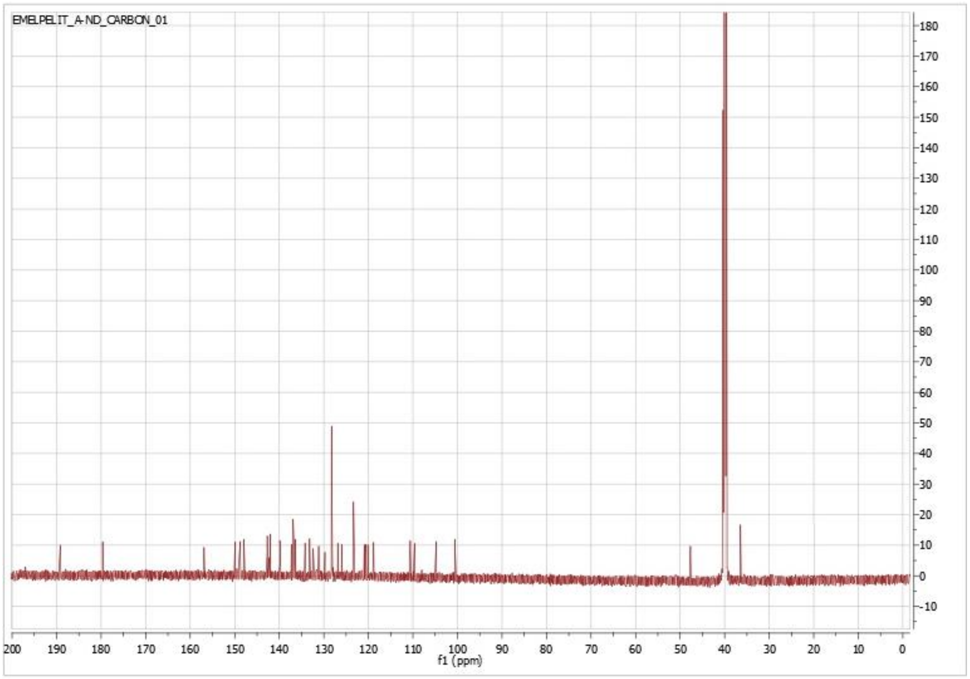

MS spectrum of compound 4b

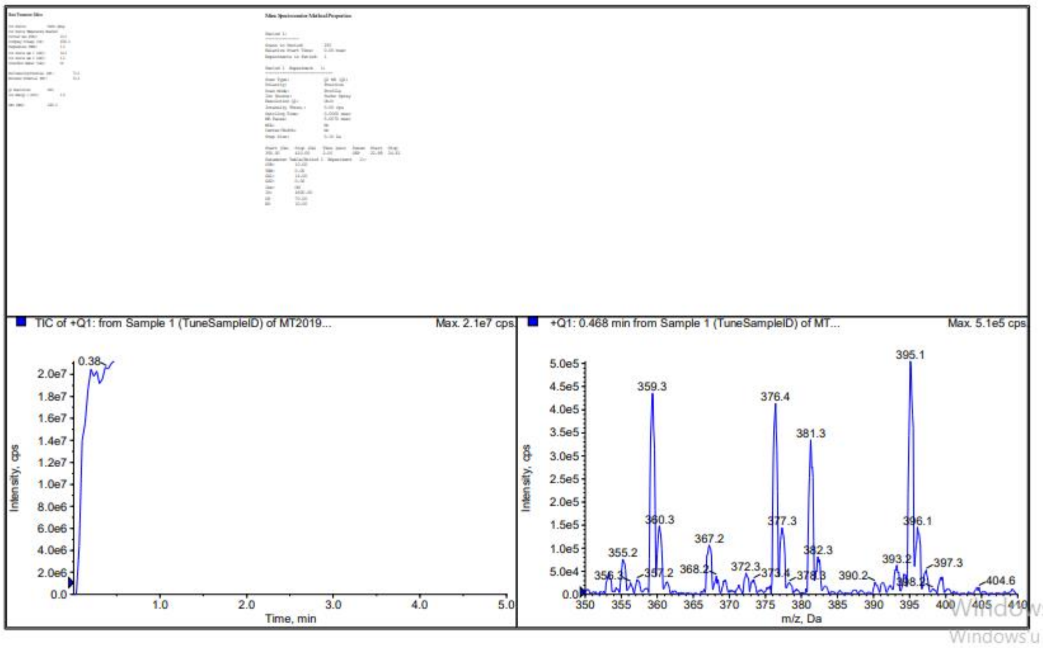

MS spectrum compound 4c

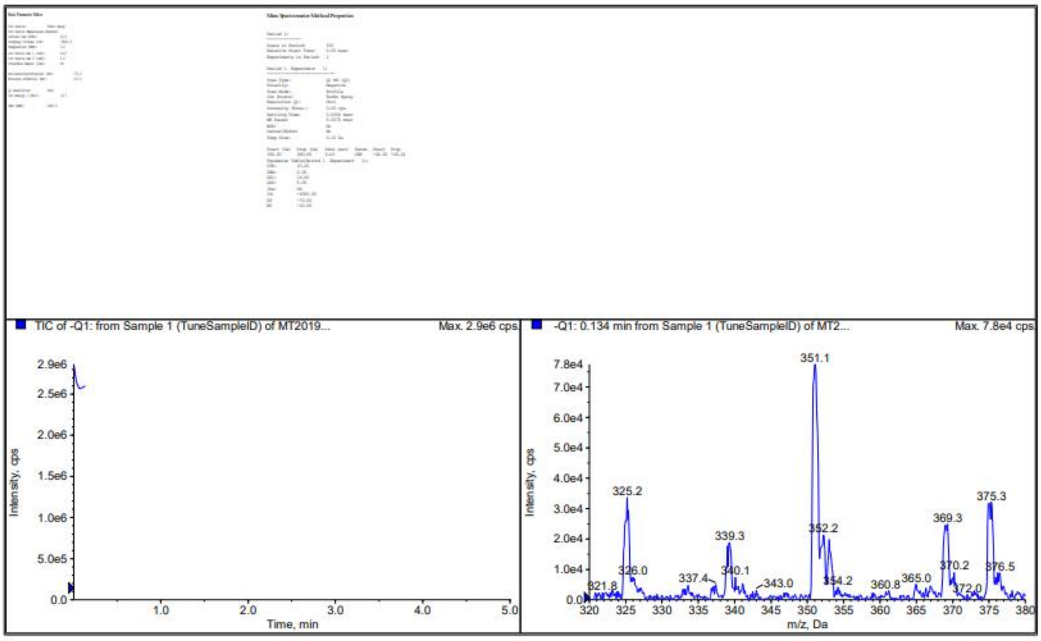

MS spectrum of compound 6a

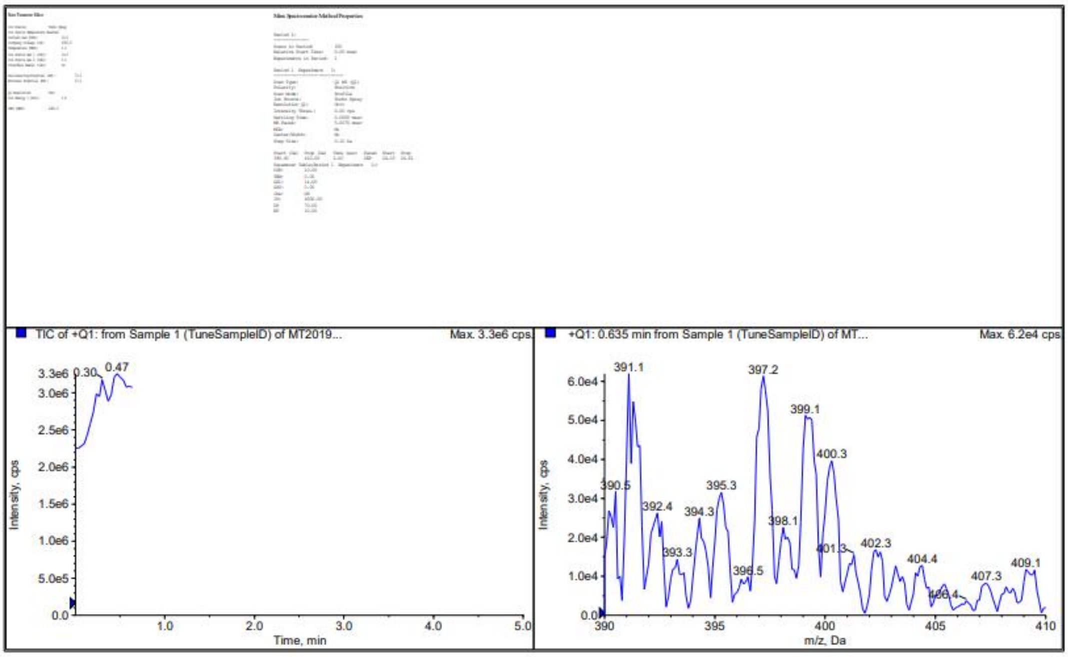

MS spectrum of compound 6d

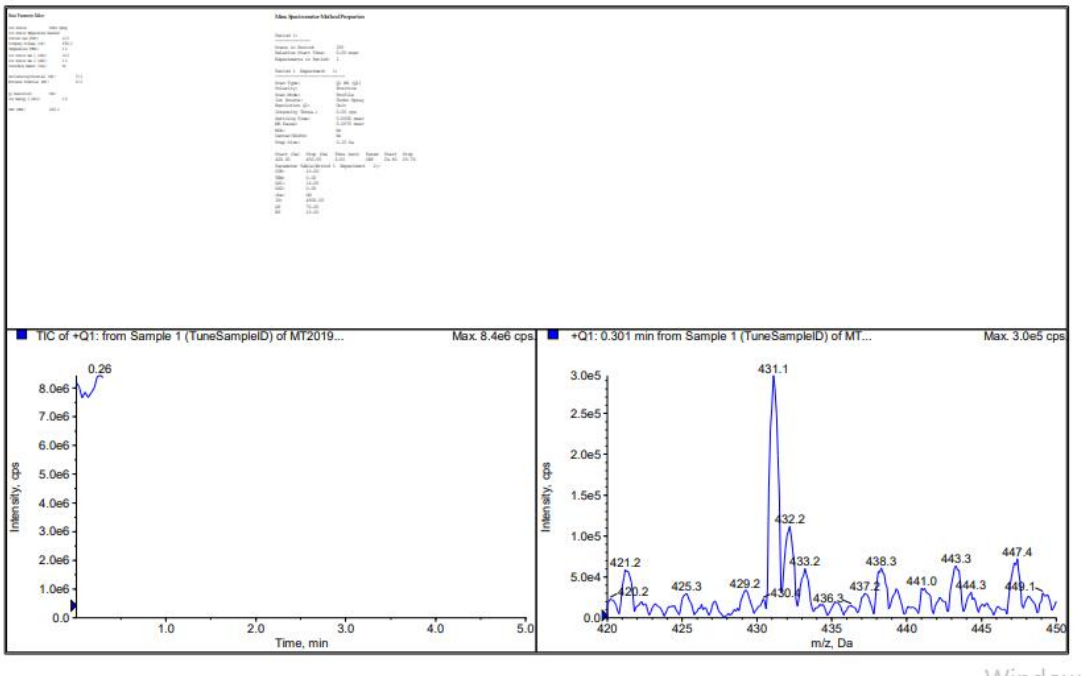

Supplement: Supplementary file 1 — Supplementary Figures. [file 41598_2023_27777_MOESM1_ESM.pdf]
